# Supplementary material for: A nanotwinned-alloy strategy enables fast sodium deposition dynamics
Source: Nat Commun. 2025 Feb 20;16:1795. doi: 10.1038/s41467-025-56957-w (PMC11842702; doi:10.1038/s41467-025-56957-w)
Supplement: Supplementary file 1 — Supplementary Information [file 41467_2025_56957_MOESM1_ESM.pdf]

Supporting Information

A Nanotwinned-Alloy Strategy Enables Fast Sodium Deposition Dynamics

Guodong Zou[1], Jinming Wang[1,2,]*, Yong Sun[1], Weihao Yang[1], Tingting Niu[1], Jinyu Li[1], Liqun Ren[3], Zhi Wei Seh[2], Qiuming Peng[1,]*

[1]*State Key Laboratory of Metastable Materials Science and Technology, Yanshan University, Qinhuangdao, 066004, P.R. China*

[2]*Institute of Materials Research and Engineering (IMRE), Agency for Science, Technology and Research (A*STAR), 2 Fusionopolis Way, Innovis #08-03, Singapore 138634, Republic of Singapore*

[3]*Laboratory of Spinal Cord Injury and Rehabilitation, Chengde Medical University, Chengde 067000, P.R.China*

Supporting Figures 1-46

Supporting Notes 1-12

Supporting Tables 1-6

Corresponding Authors: wangjinming1996@gmail.com; pengqiuming@ysu.edu.cn

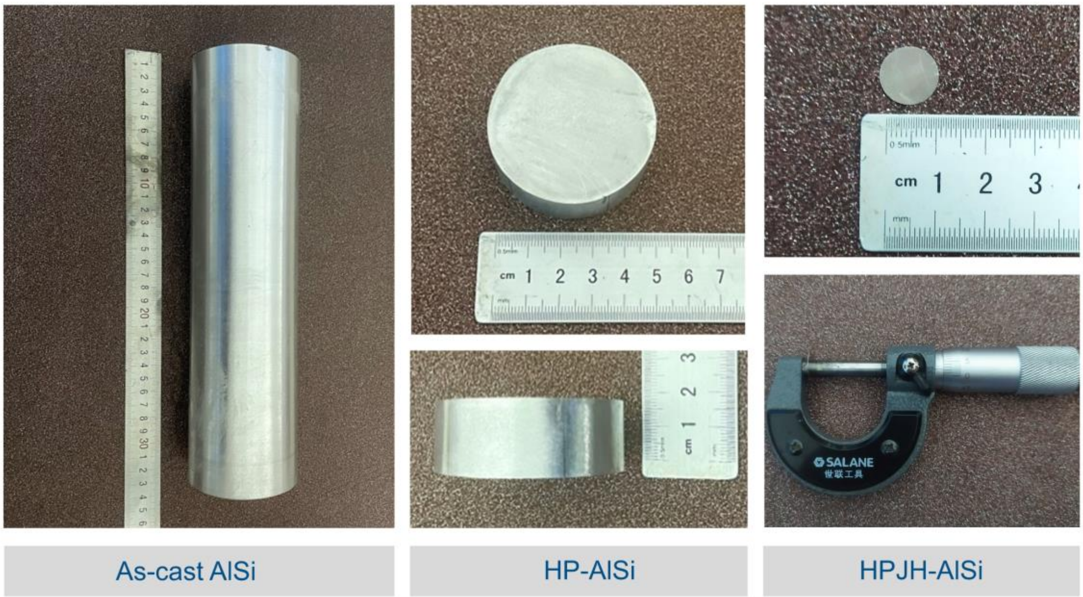

Supplementary Fig. 1. Processing and surface morphology of AlSi alloys.

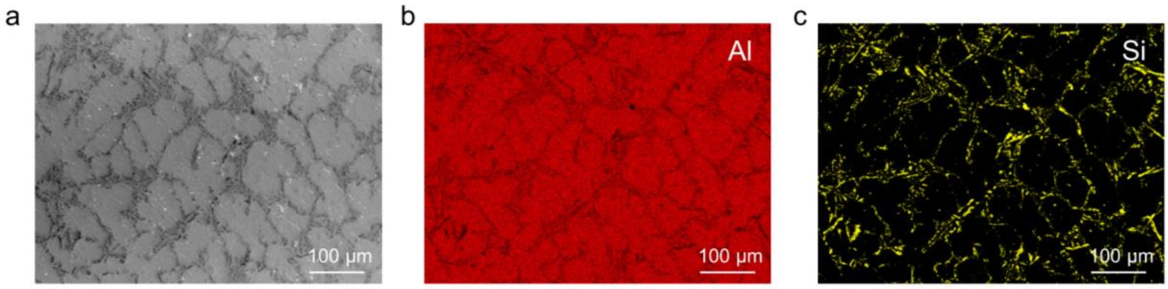

Supplementary Fig. 2. (a) SEM image of As-cast AlSi alloy. EDS images of Al (b, red color) and Si (c, yellow color) corresponding to SEM image of As-cast AlSi alloy.

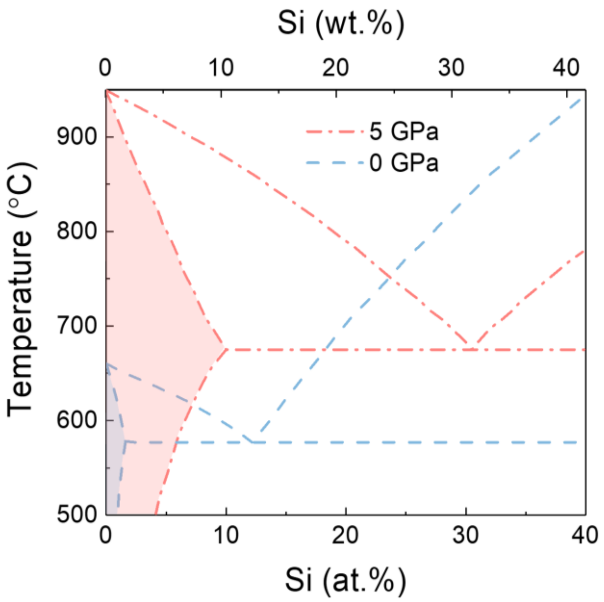

Supplementary Fig. 3. The effect of pressure on the Al-Si alloy phase diagram.

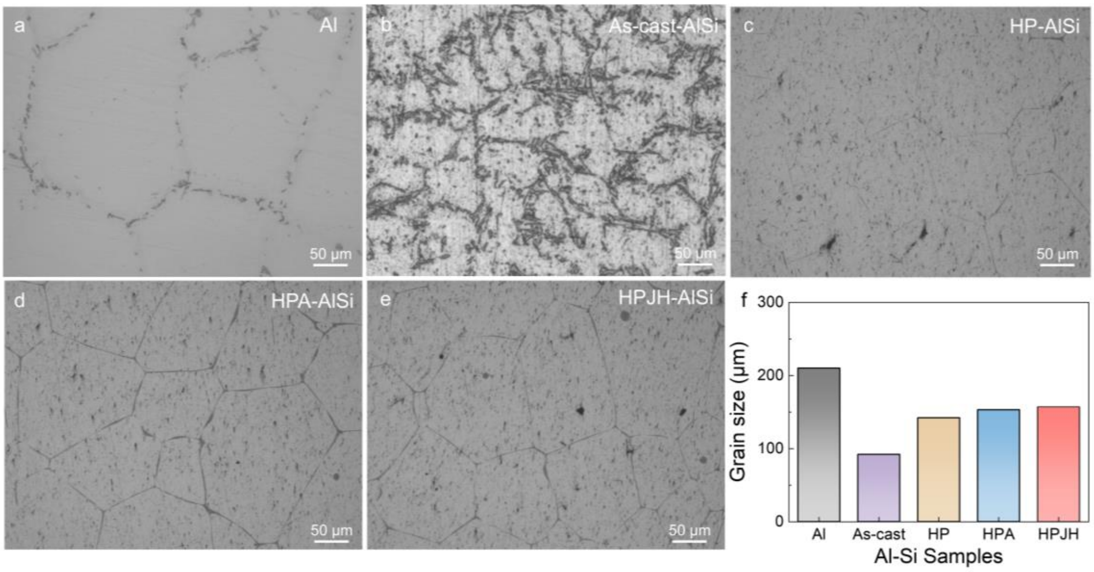

Supplementary Fig. 4. Grain size changes of different samples. Optical images of (a) Al, (b) As-cast-AlSi, (c) HP-AlSi, (d) HPA-AlSi, and (e) HPJH-AlSi. f. Comparison of grain sizes of different samples. Source data for (**f**) are provided as a Source Data file.

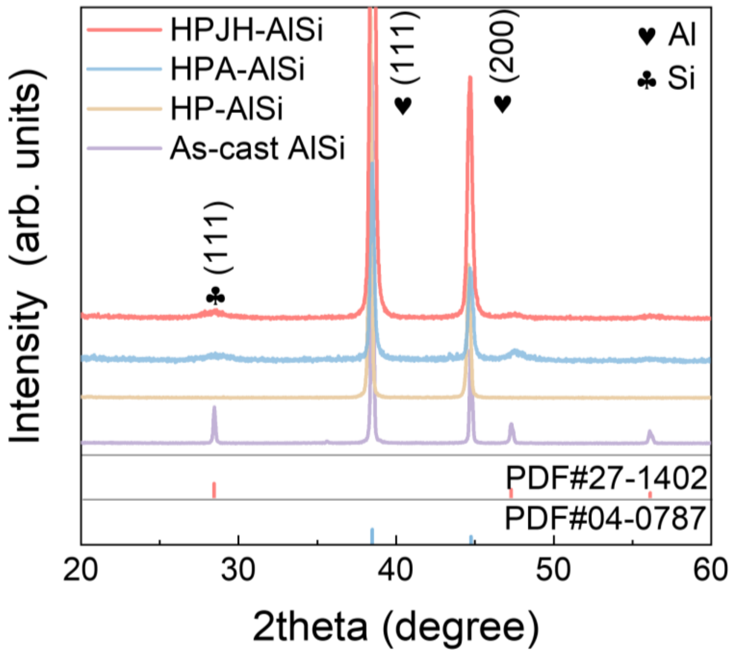

Supplementary Fig. 5. XRD patterns of the different Al-Si alloys. Source data are provided as a Source Data file.

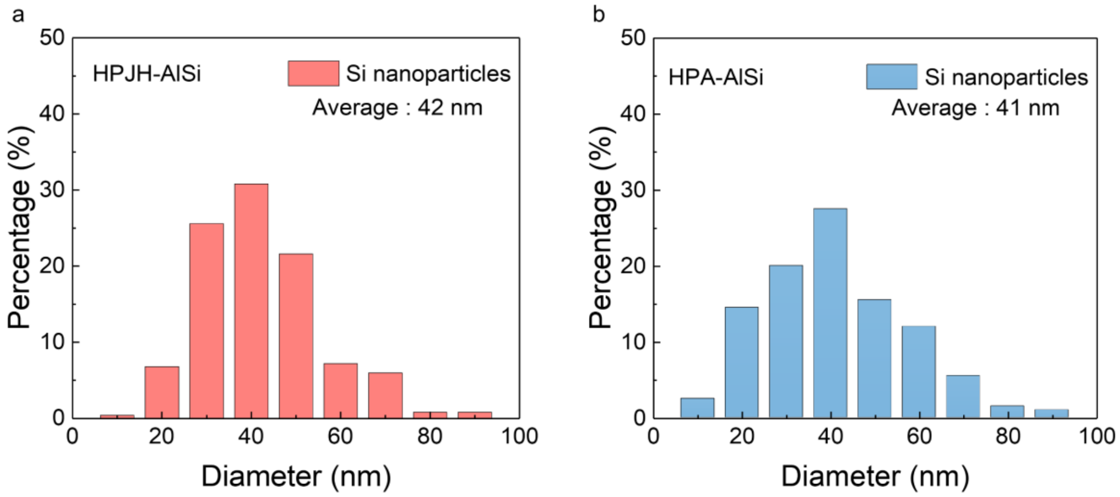

Supplementary Fig. 6. Statistical histogram of the sizes of Si nanoparticles. (a) HPJH-AlSi. (b) HPA-AlSi. Source data are provided as a Source Data file.

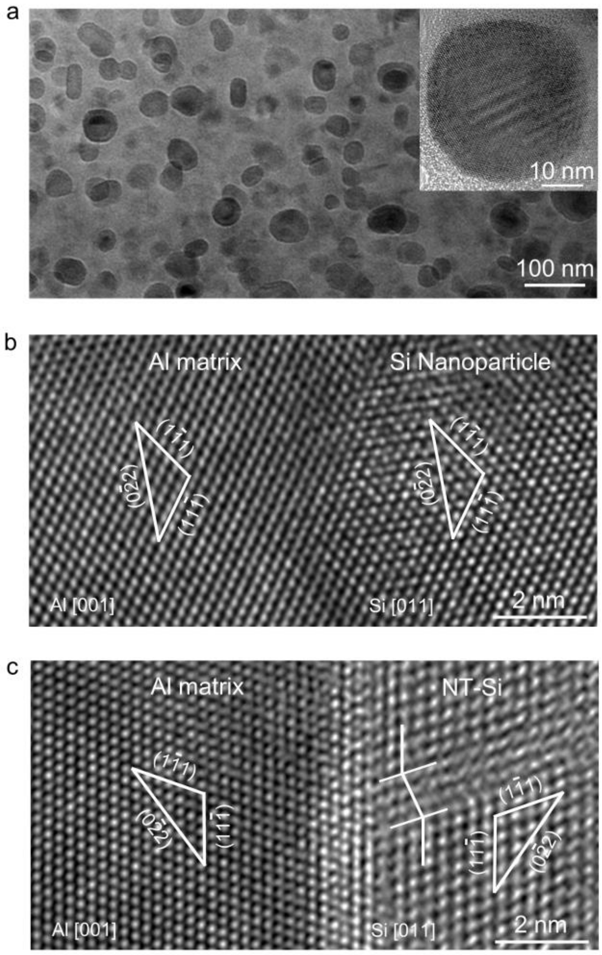

Supplementary Fig. 7. (a) TEM image of the HPA-AlSi and the inset shows the HRTEM image of Si nanocrystals. (b) HRTEM image of the interface between Al matrix and Si nanocrystals for the HPA-AlSi. (c) HRTEM image of the interface between Al matrix and NT-Si for the HPJH-AlSi.

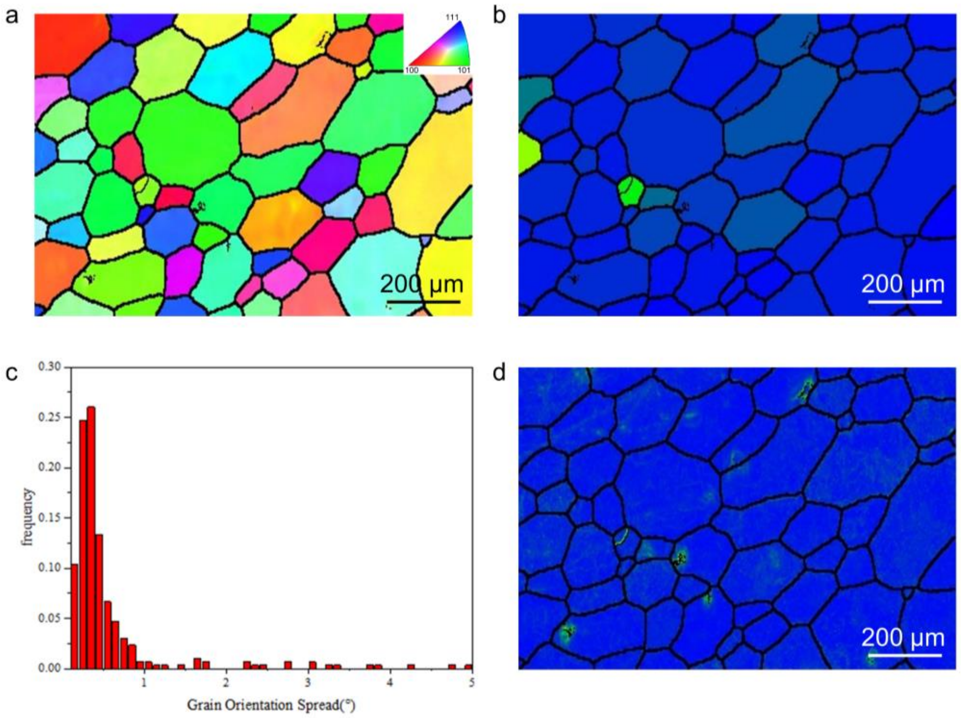

Supplementary Fig. 8. (a) Electron back scatter diffraction (EBSD) inverse pole figure (IPF) mapping of HPJH-AlSi alloy. (b) Grain orientation spread (GOS) map. (c) GOS distribution. (d) Kernel average misorientation (KAM).

.

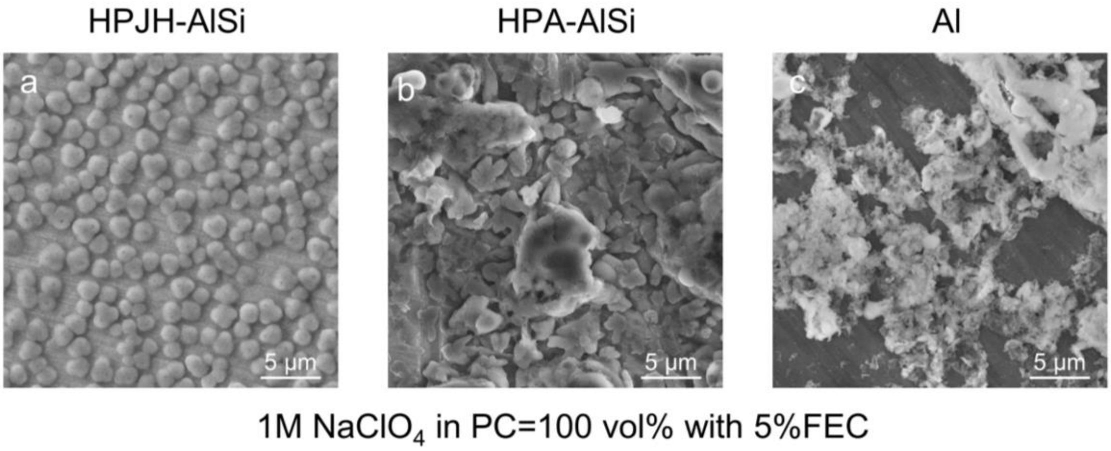

Supplementary Fig. 9. Na deposition morphology on (a) HPJH-AlSi, (b) HPA-AlSi and (c) Al collector surfaces under 1M $NaClO_4$ in propylene carbonate (PC) =100 vol% with 5% fluoroethylene carbonate (FEC) electrolyte.

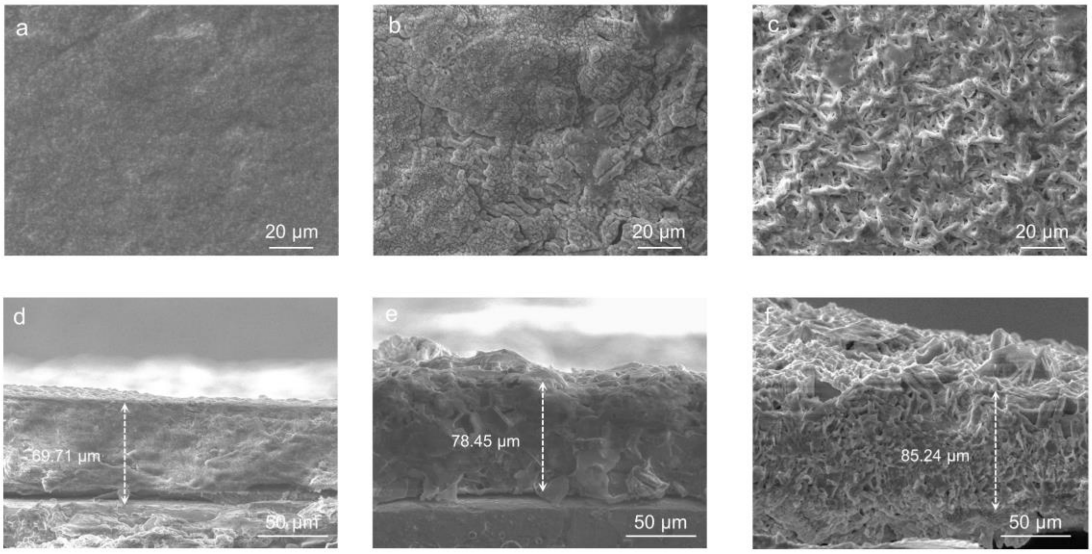

Supplementary Fig. 10. (a-c) Top view SEM images of Na deposited on HPJH-AlSi, HPA-AlSi, and Al under the current density (1 mA cm^{-2}), with capacities (7 mAh cm^{-2}). (d-f) Cross-section SEM images of Na deposited on HPJH-AlSi, HPA-AlSi, and Al under the current density (1 mA cm^{-2}), with capacities (7 mAh cm^{-2}).

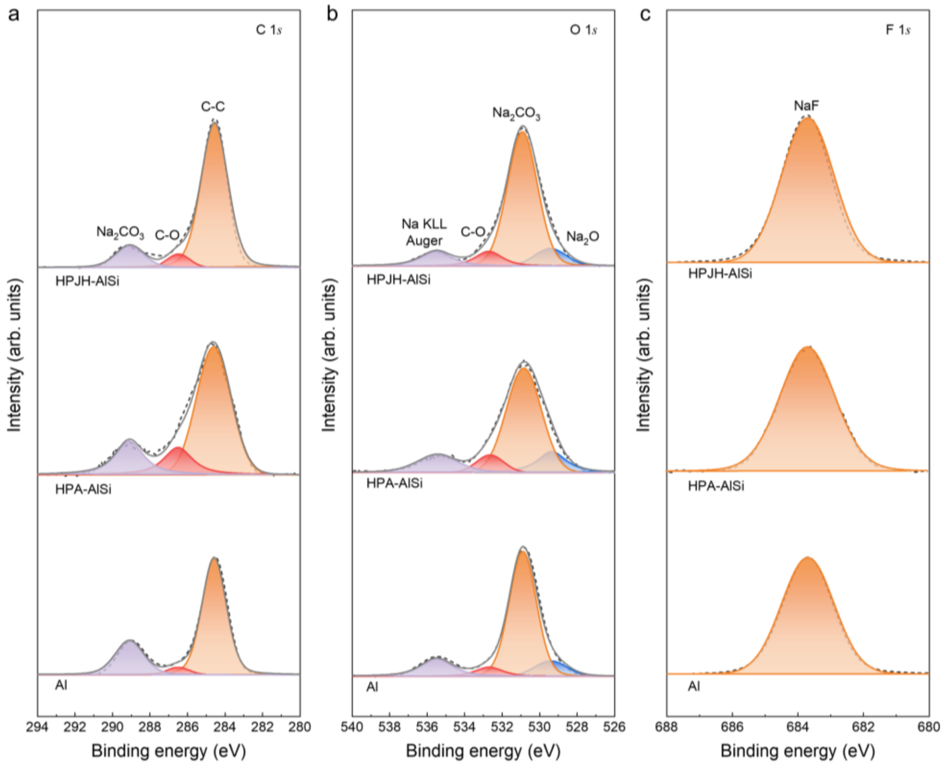

Supplementary Fig. 11. XPS profiles of the HPJH-AlSi, HPA-AlSi and Al electrodes after deposition. (a) C 1*s* spectrum, (b) O 1*s* spectrum, and (c) F 1*s* spectrum. The HPJH-AlSi, HPA-AlSi and Al collectors were assembled with Na metal foils and plated/stripped at a current density of 1 mA cm^{-2} and a capacity of 1 mAh cm^{-2}. All the tests were measured under 25 °C using the 1 M $NaPF_6$ in diglyme =100 vol% electrolyte. Source data are provided as a Source Data file.

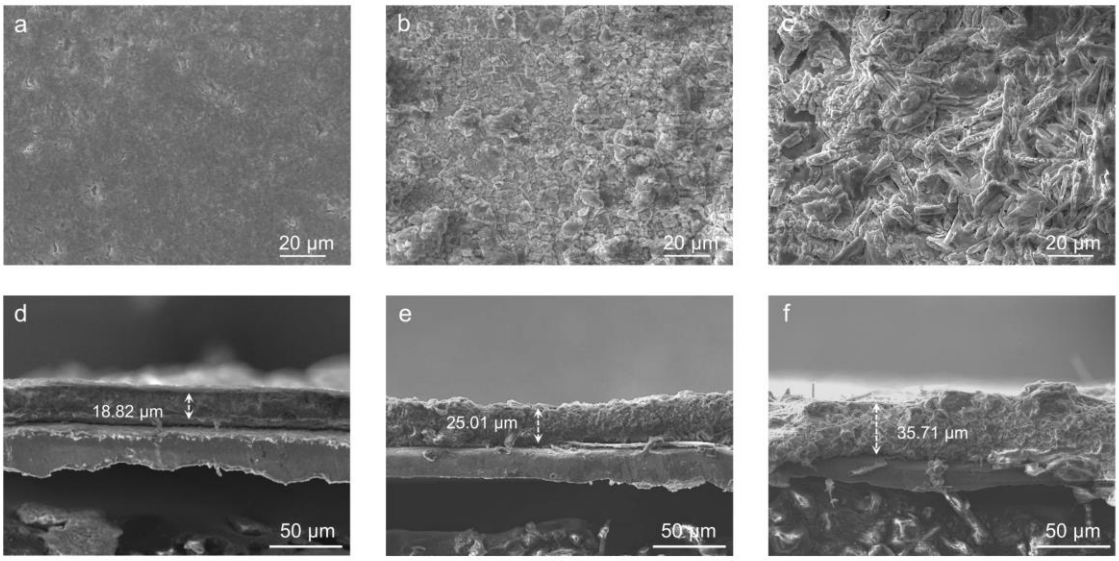

Supplementary Fig. 12. (a-c) Top view SEM images of Na deposited on HPJH-AlSi, HPA-AlSi, and Al with the capacities of 2 mAh cm^{-2}. (d-f) Cross-section SEM images of Na deposited on HPJH-AlSi, HPA-AlSi, and Al with the capacities of 2 mAh cm^{-2}. The HPJH-AlSi, HPA-AlSi and Al collectors were assembled with Na metal foils and plated/stripped at a current density of 1 mA cm^{-2}. All the tests were measured under 25 °C using the 1 M $NaPF_6$ in diglyme =100 vol% electrolyte.

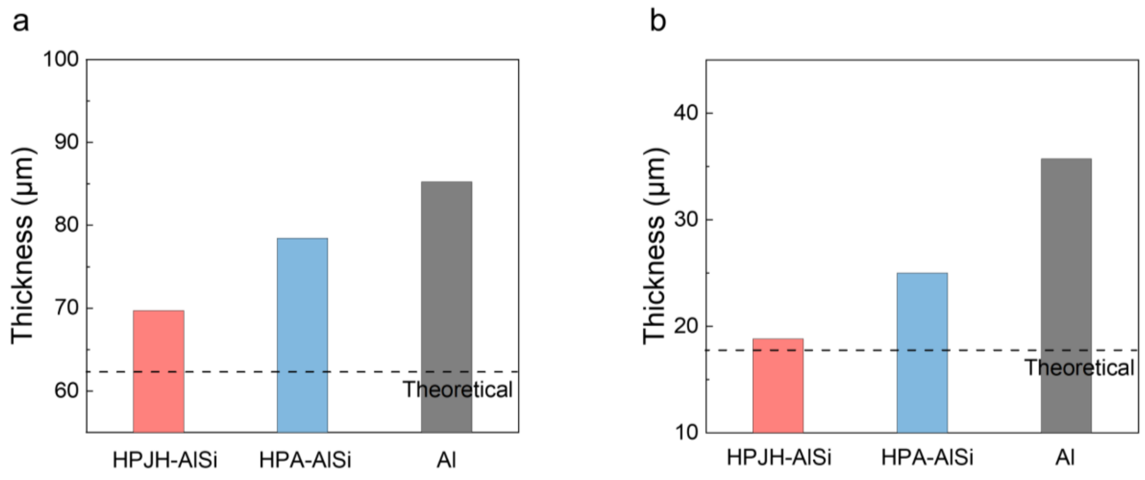

Supplementary Fig. 13. (a) Summarized average thickness data of Na deposited on HPJH-AlSi, HPA-AlSi and Al collector under 1 mA cm^{-2}, 7 mAh cm^{-2}. (b) Summarized average thickness data of Na deposited on HPJH-AlSi, HPA-AlSi and Al collector under 2 mA cm^{-2}, 2 mAh cm^{-2}. Source data are provided as a Source Data file.

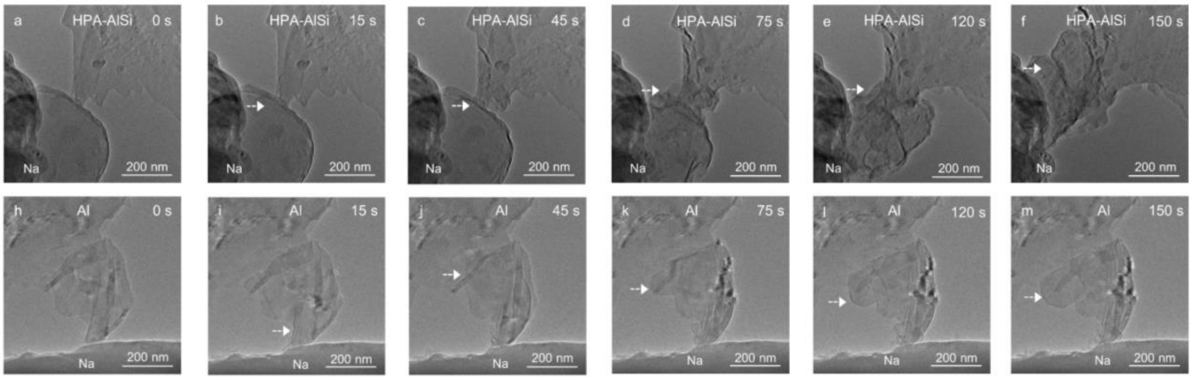

Supplementary Fig. 14. (a-f) Time lapse TEM images of Na metal deposition on HPA-AlSi collector. (h-m) Time lapse TEM images of Na metal deposition on Al collector. The nano cells were plated at a current density of 3 mA cm^{-2} and measured under 25 ℃.

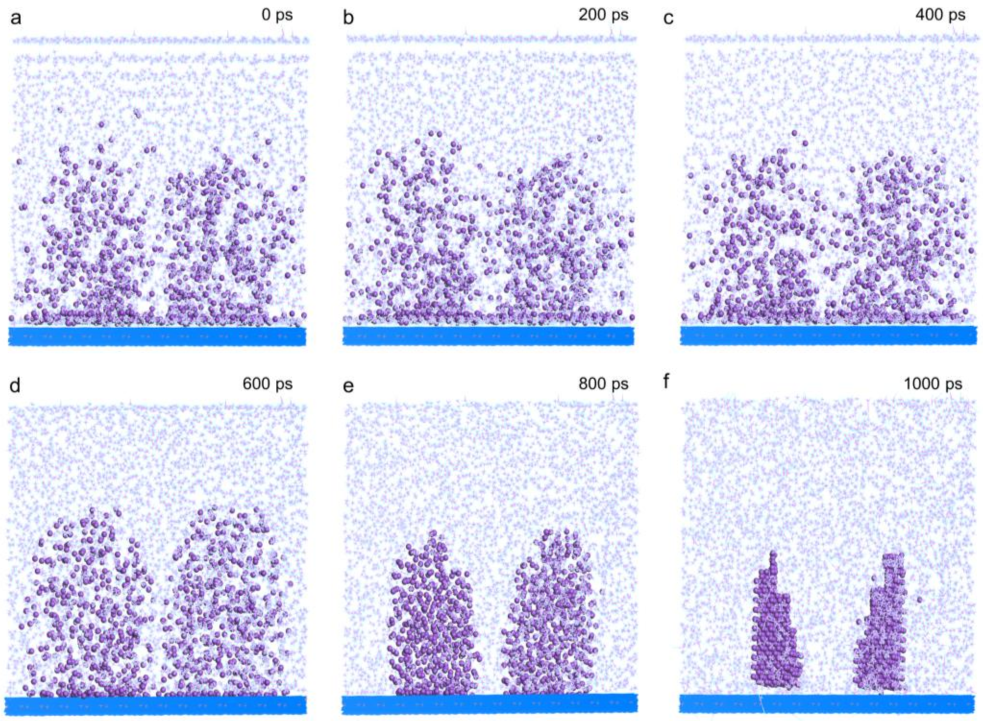

Supplementary Fig. 15. (a-f) The temporal evolution of Na deposition on HPA-AlSi obtained with MD simulations. The MD data are provided at https://doi.org/10.24435/materialscloud:r1-e1.

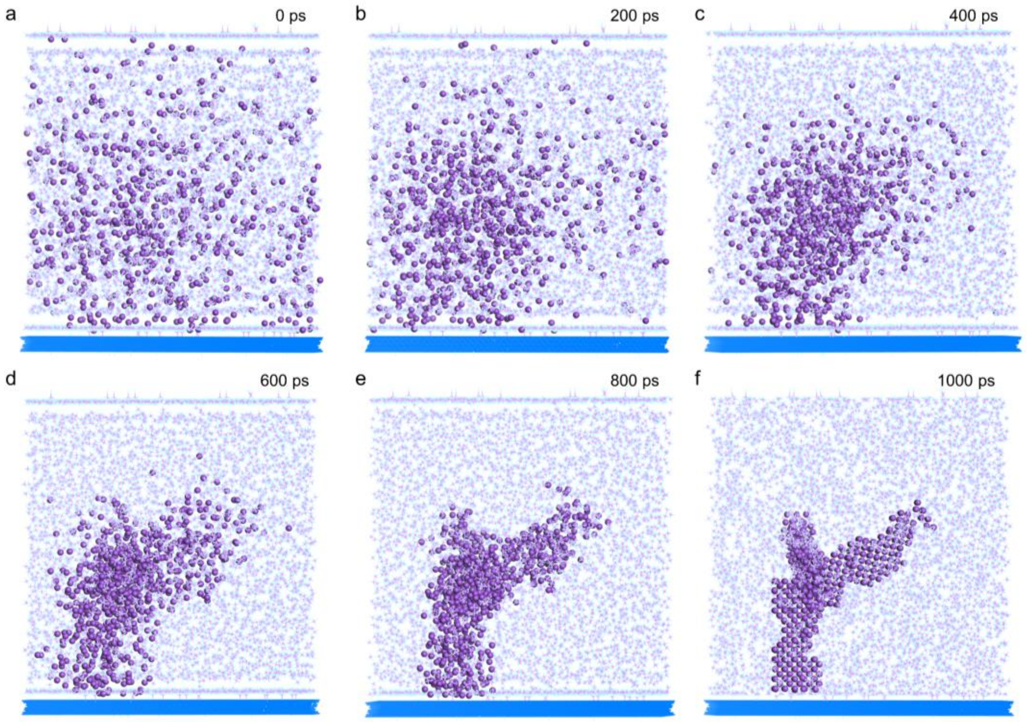

Supplementary Fig. 16. (a-f) The temporal evolution of Na deposition on Al obtained with MD simulations. The MD data are provided at https://doi.org/10.24435/materialscloud:r1-e1.

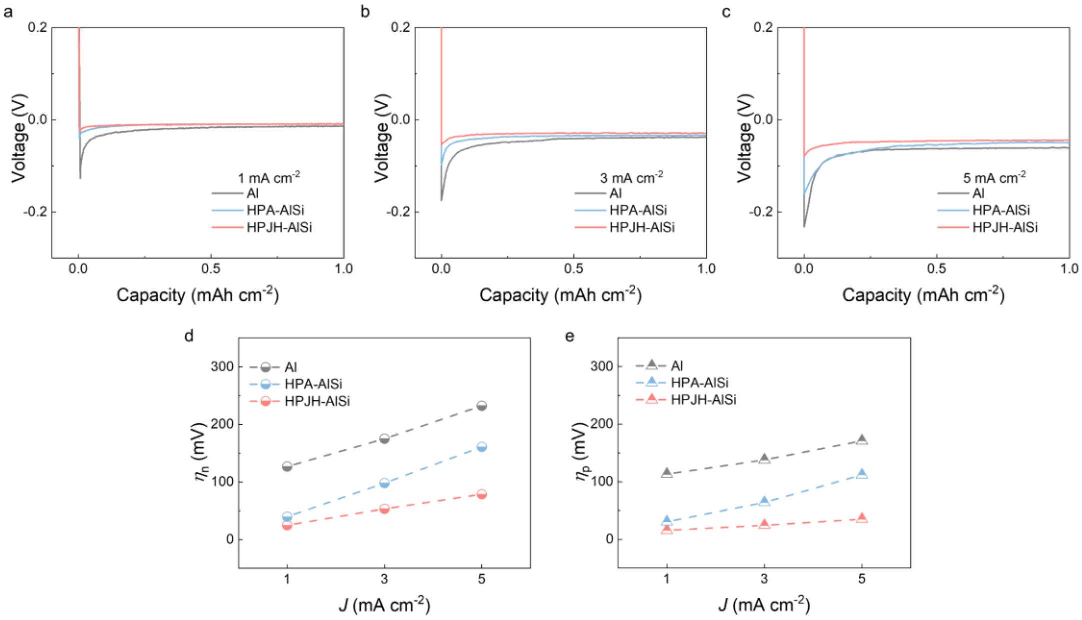

Supplementary Fig. 17. (a-c) Voltage profiles during deposition at different current densities. (d, e) Nucleation overpotential (η_n) and plating overpotential (η_p) versus current density (J), respectively. The nucleation overpotential (η_n), denoted as the minimum voltage, and the plating overpotential (η_p), corresponding to the plateau voltage[1]. Source data are provided as a Source Data file.

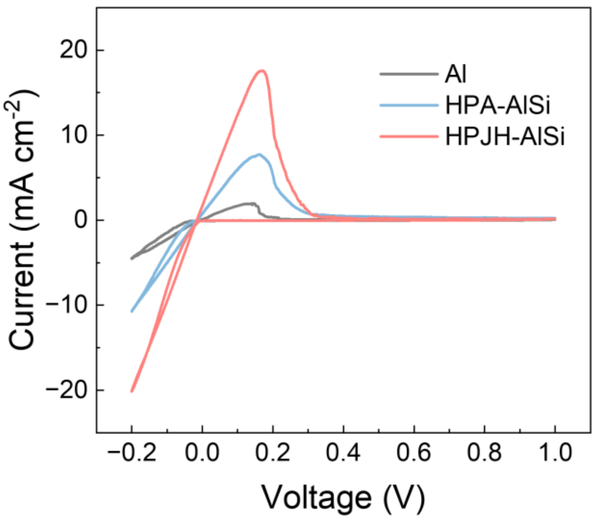

Supplementary Fig. 18. Cyclic voltammograms for Na deposition/stripping over different collectors at a scan rate of 5 mV s^{-1}. Source data are provided as a Source Data file.

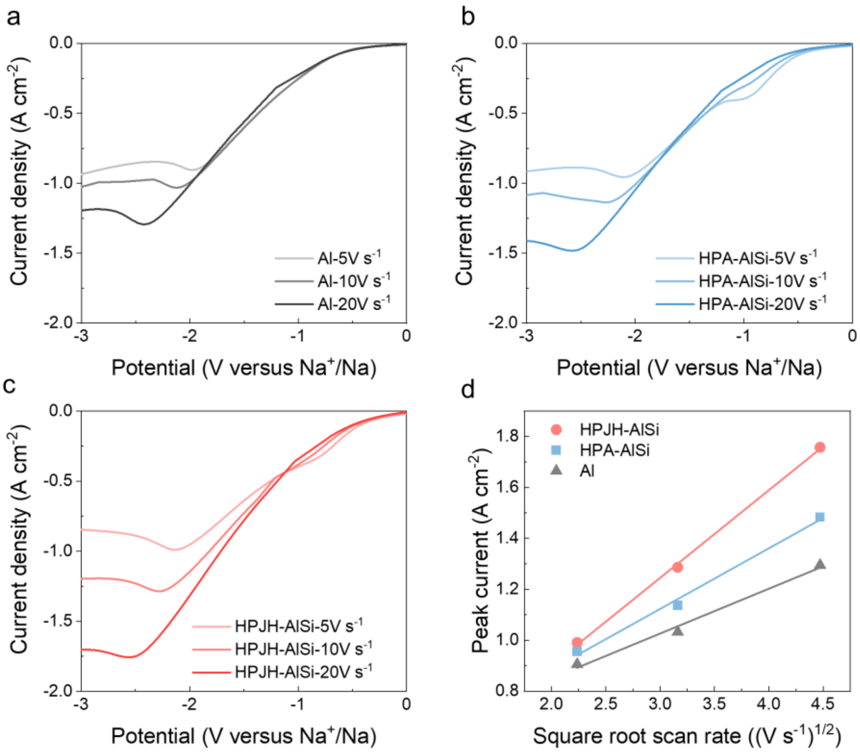

Supplementary Fig. 19. (a-c) LSV profiles of Na electrodeposition with a fast scan rate from 5 to 20 V s^{-1}. (d) The dependence of the peak current on the square root of the scan rate. Source data are provided as a Source Data file.

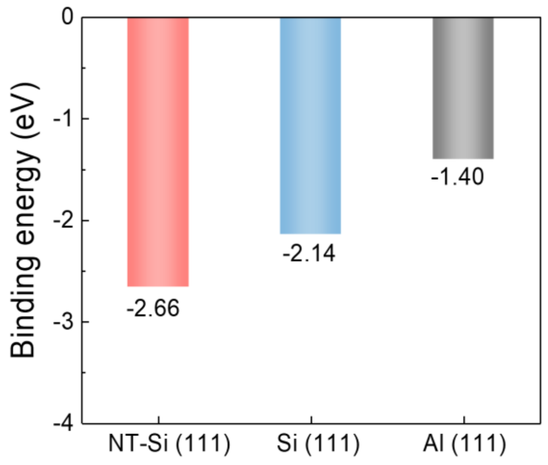

Supplementary Fig. 20. Calculation of the binding energies of different crystal facets with Na atoms.

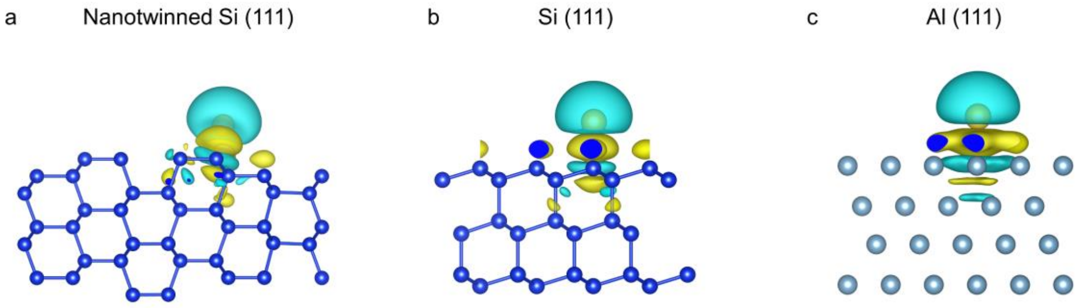

Supplementary Fig. 21. The three-dimensional charge density differences of (a) NT-Si (111), (b) Si (111), and (c) Al (111) with Na adsorbed. Light blue spheres represents Al atoms, dark blue spheres represents Si atoms and yellow spheres represents Na atoms. Cyan area represents charge depletion, and yellow area represents charge accumulation. The DFT data are provided at https://doi.org/10.24435/materialscloud:r1-e1.

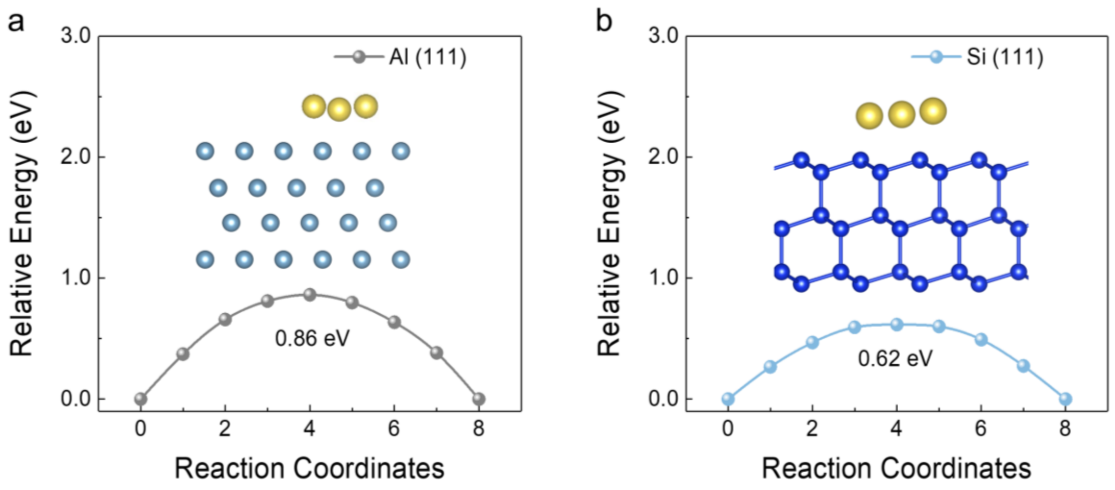

Supplementary Fig. 22. The diffusion pathway and calculated energy profile of Na along the diffusion path of Na on (a) Al (111) and (b) Si (111). Light blue spheres represents Al atoms, dark blue spheres represents Si atoms and yellow spheres represents Na atoms. The DFT data are provided at https://doi.org/10.24435/materialscloud:r1-e1. Source data are provided as a Source Data file.

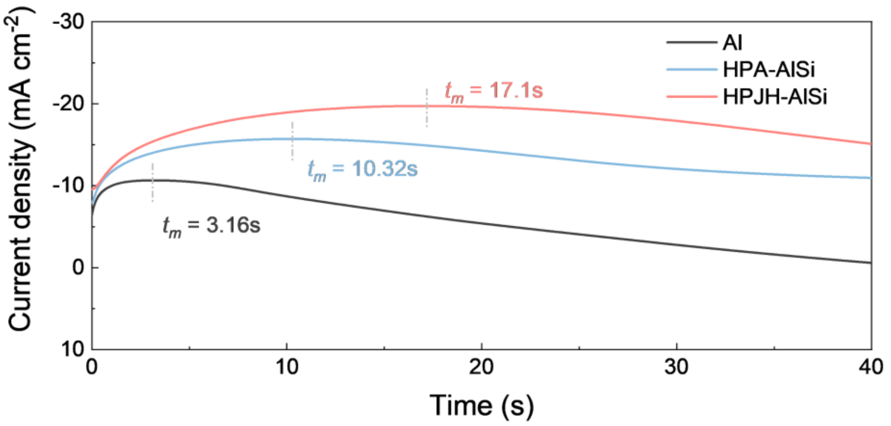

Supplementary Fig. 23. Current-time transients obtained at predetermined potentials. j_{m}: peak current, t_{m}: time needed to achieve the peak current. Source data are provided as a Source Data file.

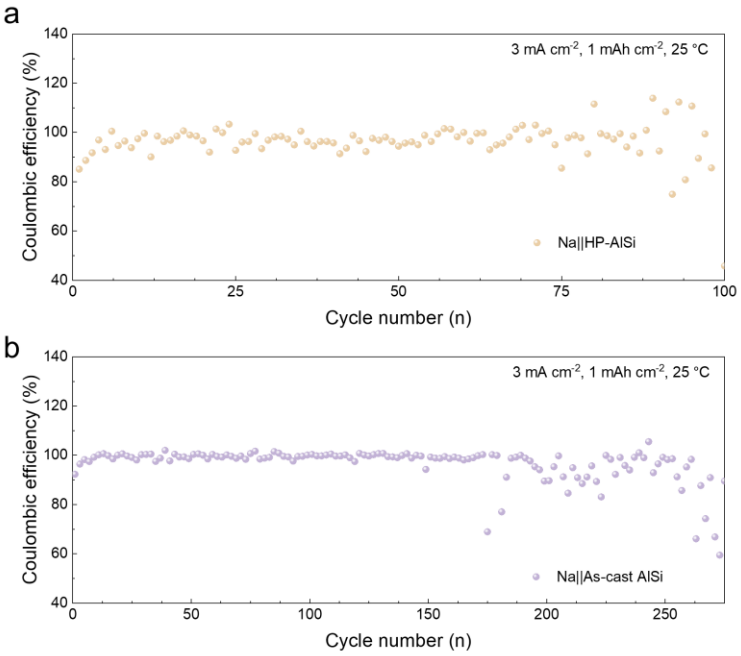

Supplementary Fig. 24. Coulombic efficiencies of Na plating/stripping on the (a) HP-AlSi and (b) As-cast AlSi at current densities of 3 mA cm^{-2} and areal capacities of 1 mAh cm^{-2}. All the tests were measured at 25 ℃ using the 1 M $NaPF_6$ in diglyme =100 vol% electrolyte. Source data are provided as a Source Data file.

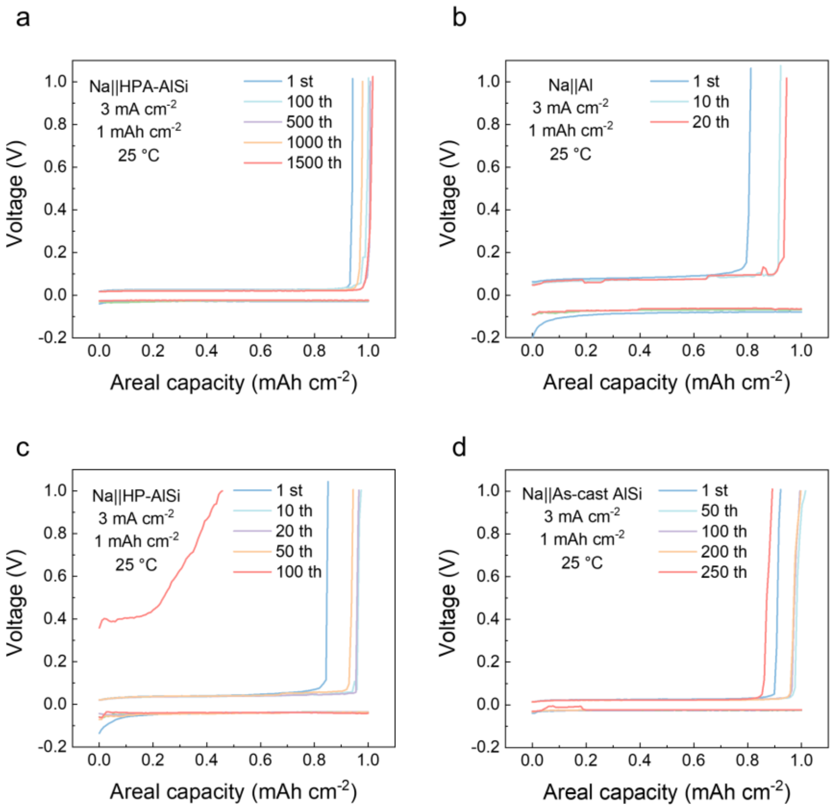

Supplementary Fig. 25. Plating/stripping stability of the half cell. Voltage-capacity profiles of (a) HPA-AlSi, (b) Al, (c) HP-AlSi and (d) As-cast AlSi at 3 mA cm^{-2} and 1 mAh cm^{-2}. All the tests were measured at 25 ℃ using the 1 M $NaPF_6$ in diglyme =100 vol% electrolyte. Source data are provided as a Source Data file.

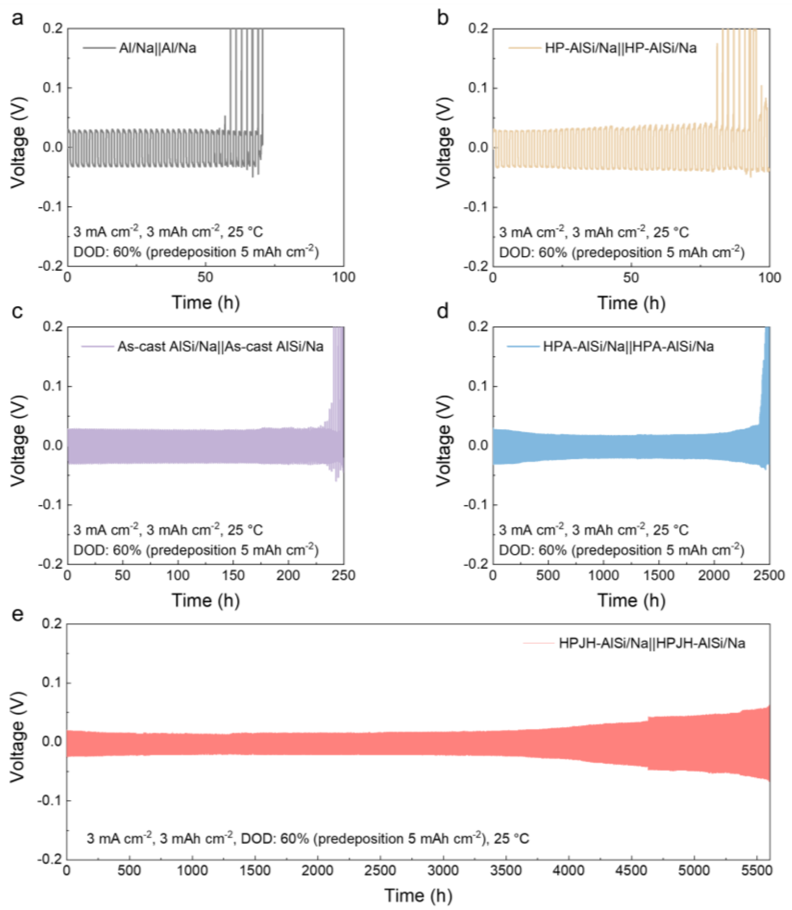

Supplementary Fig. 26. Voltage profiles of symmetric cells assembled with (a) Al, (b) HP-AlSi, (c) As-cast AlSi, (d) HPA-AlSi and (e) HPJH-AlSi at 3 mA cm^{-2} and 3 mAh cm^{-2}. The depth of discharge (DOD) is identified by: DOD= (Na deposition/stripping capacity)/(Na predeposition capacity)*100%. All the tests were measured at 25 ℃ using the 1 M $NaPF_6$ in diglyme =100 vol% electrolyte. Source data are provided as a Source Data file.

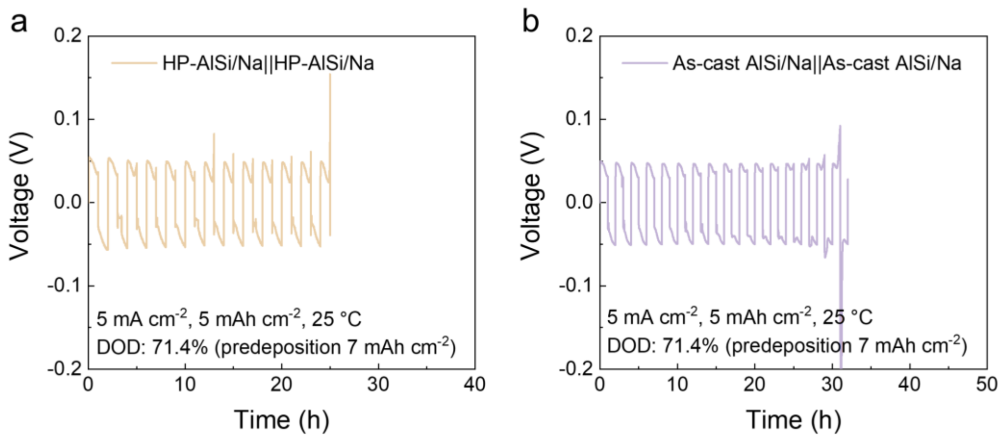

Supplementary Fig. 27. Voltage profiles of symmetric cells assembled with (a) HP-AlSi and (b) As-cast AlSi at 5 mA cm^{-2} and 5 mAh cm^{-2}. All the tests were measured at 25 ℃ using the 1 M $NaPF_6$ in diglyme =100 vol% electrolyte. Source data are provided as a Source Data file.

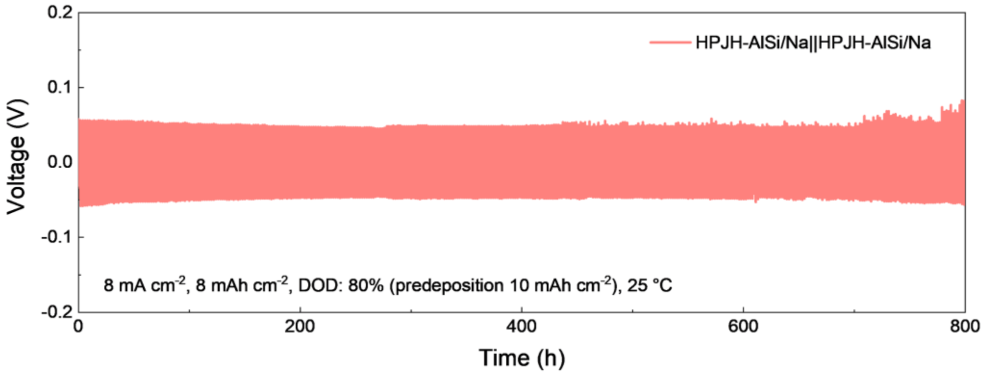

Supplementary Fig. 28. Voltage profiles of symmetric cells assembled with HPJH-AlSi at 80 % DOD, 8 mA cm^{-2} and 8 mAh cm^{-2}. All the tests were measured at 25 ℃ using the 1 M $NaPF_6$ in diglyme =100 vol% electrolyte. Source data are provided as a Source Data file.

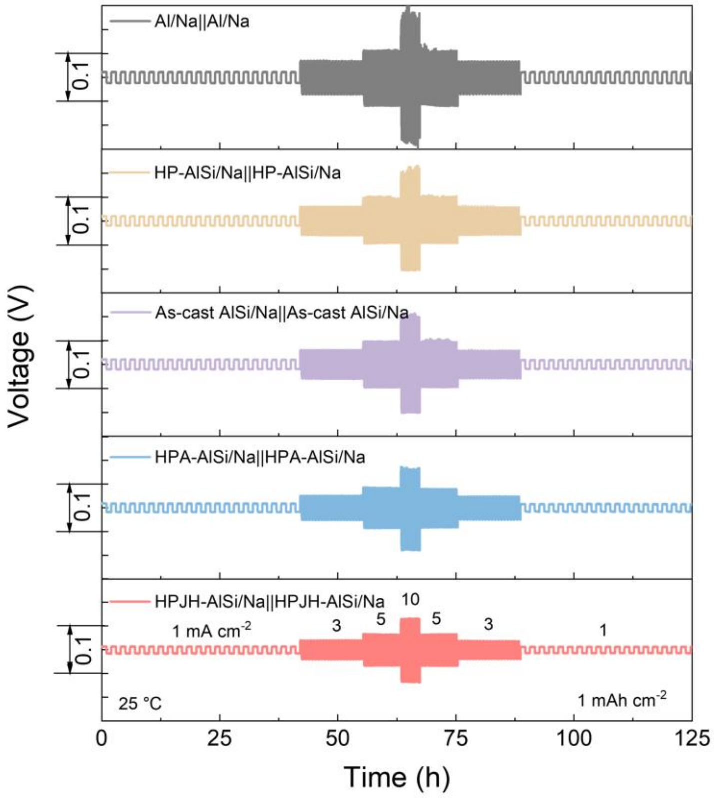

Supplementary Fig. 29. Rate capability voltage profiles of symmetric cells at various current densities. All the tests were measured at 25 ℃ using the 1 M $NaPF_6$ in diglyme =100 vol% electrolyte. Source data are provided as a Source Data file.

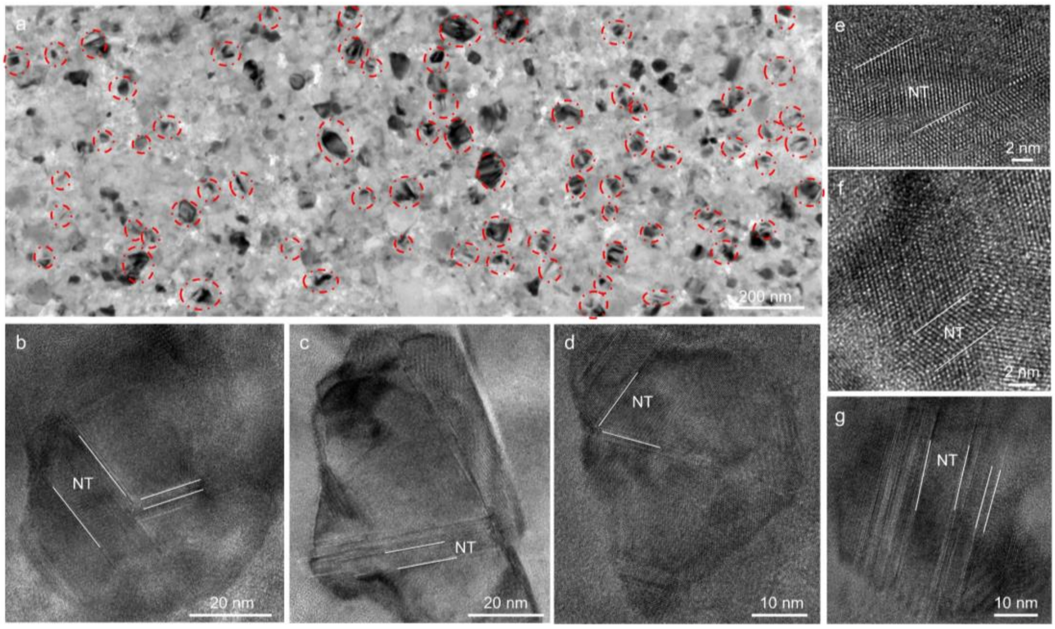

Supplementary Fig. 30. (a) TEM characterization of HPJH-AlSi after electrochemical testing. (b-g) HRTEM images in the HPJH-AlSi with nanotwin. The HPJH-AlSi collector was assembled with Na metal foils and plated/stripped at a current density of 5 mA cm^{-2} and a capacity of 5 mAh cm^{-2} for 10 cycles. All the tests were measured at 25 ℃ using the 1 M $NaPF_6$ in diglyme =100 vol% electrolyte.

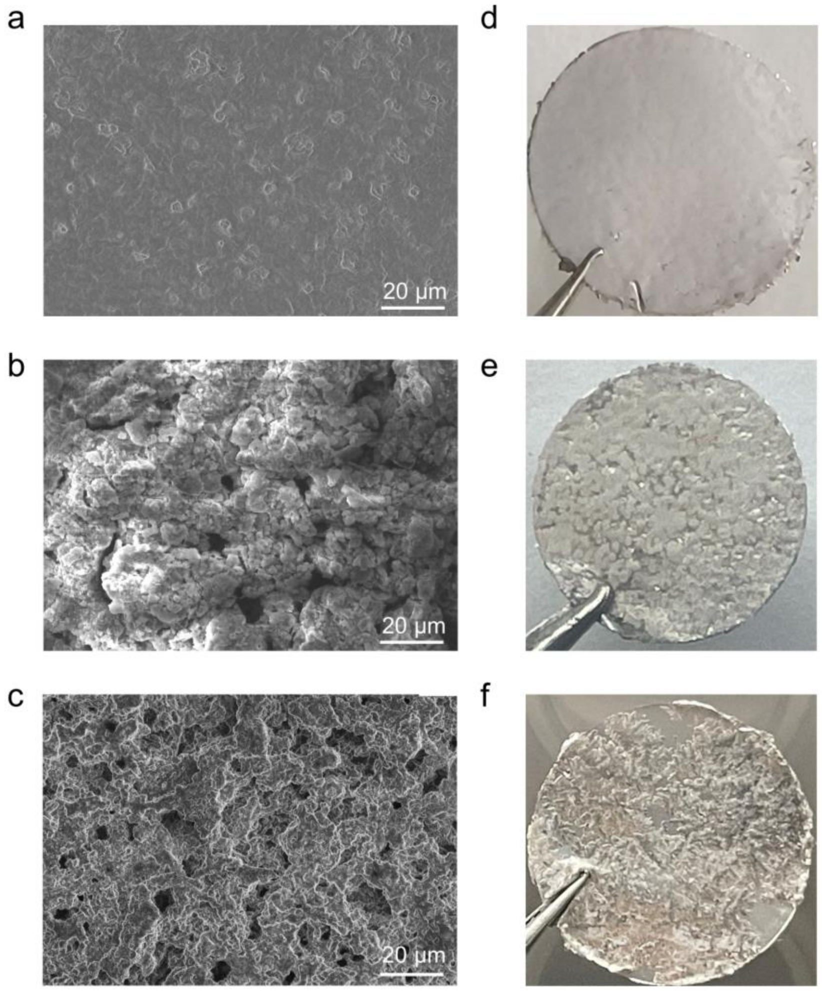

Supplementary Fig. 31. SEM images (a-c) and optical images (d-f) of HPJH-AlSi/Na (a, d), HPA-AlSi/Na (b, e), and Al/Na (c, f) electrodes after Na plating/stripping at a current density of 5 mA cm^{-2} and a capacity of 5 mAh cm^{-2} for 10 cycles. All the tests were measured at 25 ℃ using the 1 M $NaPF_6$ in diglyme =100 vol% electrolyte.

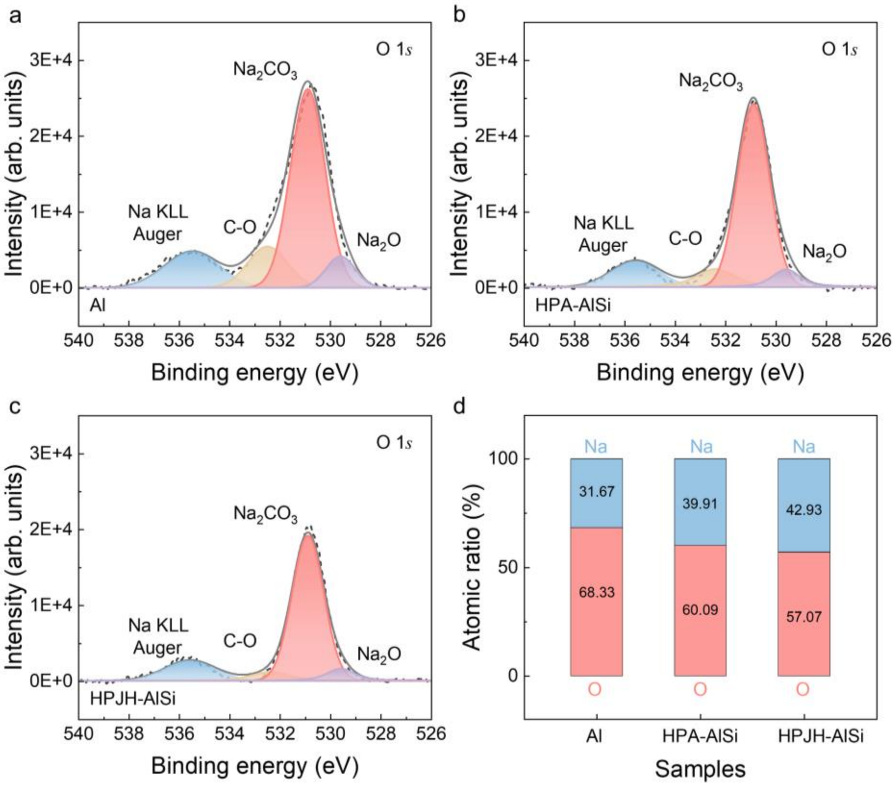

Supplementary Fig. 32. (a-c) XPS patterns of high-resolution O 1*s* of different electrodes after 10 cycles. (d) The relative ratio of Na versus O in surface layer of three electrodes according the XPS data. Source data are provided as a Source Data file.

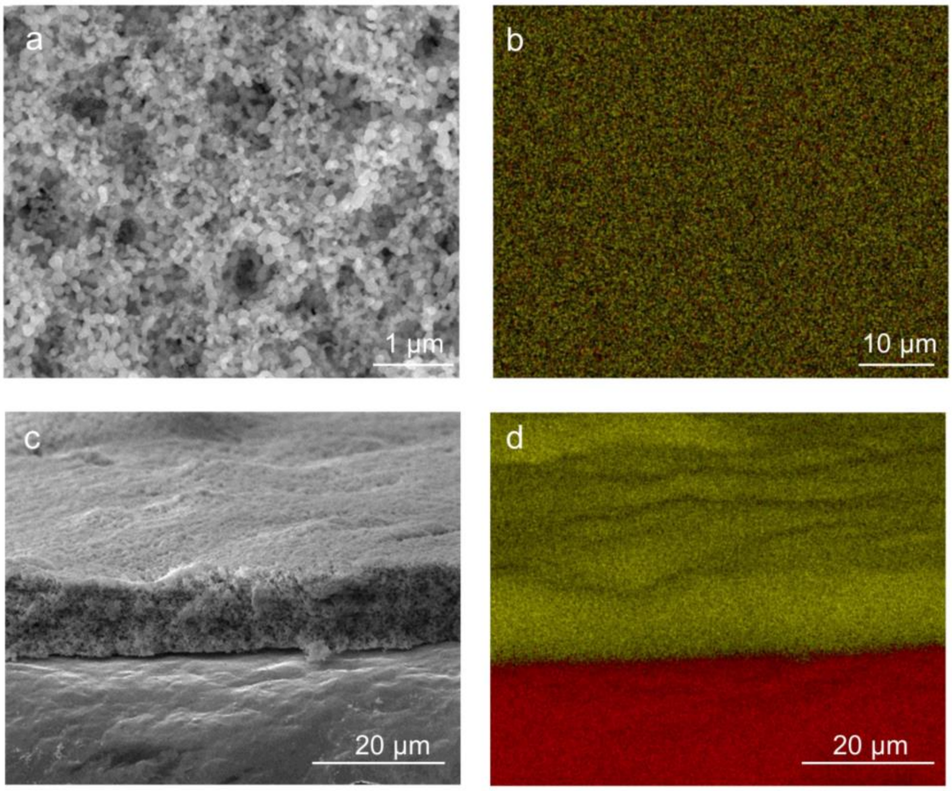

Supplementary Fig. 33. (a, b)Top view of Si-coated-Al and corresponding energy dispersive X-ray spectroscopy (EDS) mapping, (c, d) Side view of Si-coated-Al and corresponding EDS mapping. The Si coating consists of nano-scale Si with a coating thickness of about 10 μm; furthermore, in EDS mapping, yellow color represents Si and red color represents Al.

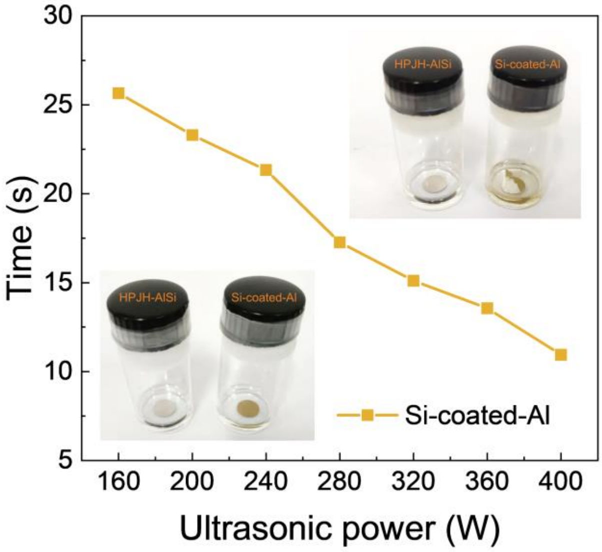

Supplementary Fig. 34. Stability on collector in electrolyte. Source data are provided as a Source Data file.

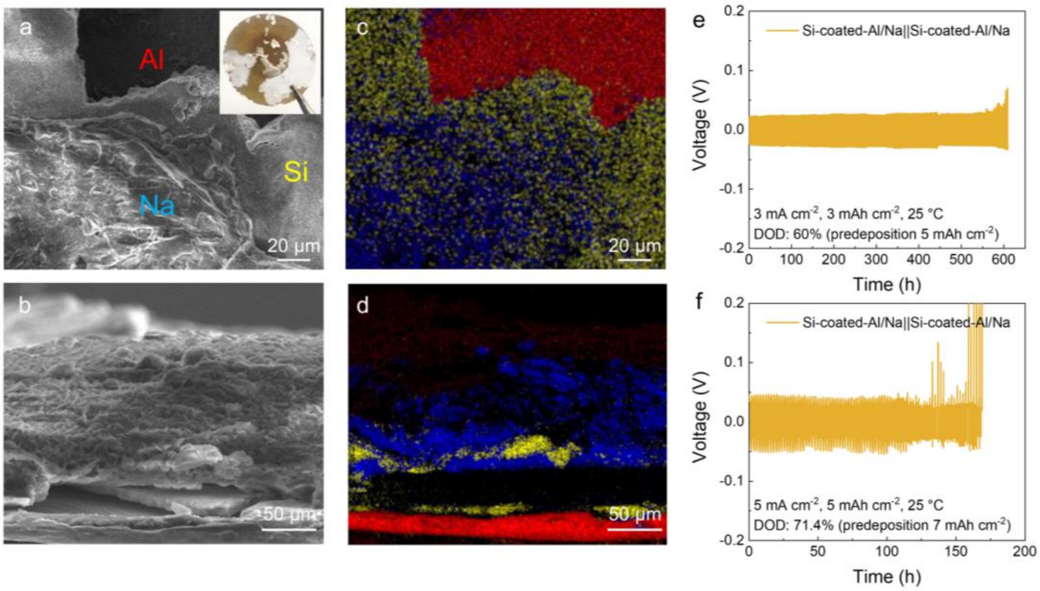

Supplementary Fig. 35. (a-d) SEM images and corresponding EDS spectra of (a, c) top view and (b, d) cross-section of Si-coated-Al electrode after Na plating/stripping. Red color represents Al, yellow color represents Si and blue color represents Na. e, f Voltage profiles of symmetric cells assembled with Si-coated-Al e at 3 mA cm^{-2}, 3 mAh cm^{-2} (e), and 5 mA cm^{-2}, 5 mAh cm^{-2} (f). All the tests were measured at 25 ℃ using the 1 M $NaPF_6$ in diglyme =100 vol% electrolyte. Source data for (e and f) are provided as a Source Data file.

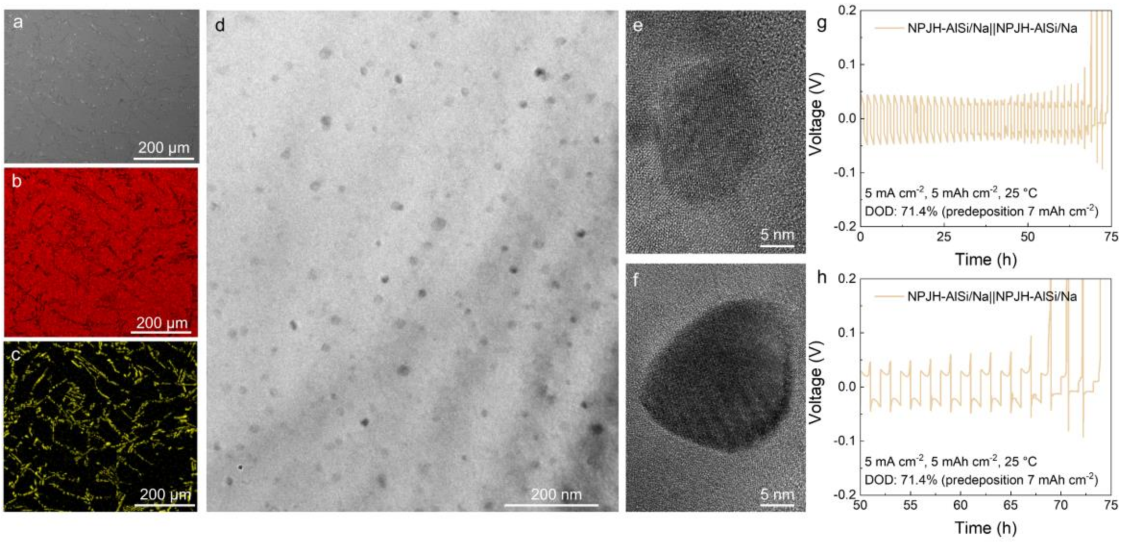

Supplementary Fig. 36. (a-c) SEM image of the AlSi alloy prepared by normal pressure solid solution process combined with joule heating (NPJH), and the corresponding element analysis spectrum of Al (b, red color) and Si (b, yellow color). (d) TEM image of the NPJH-AlSi alloy. (e, f) HRTEM images of the NPJH-AlSi alloy. (g) Voltage profiles of the NPJH-AlSi alloy at 5 mA cm^{-2}, 5 mAh cm^{-2}. (h) Magnified view of selected cycle times between 50 and 75 h for the symmetric cell assembled with the NPJH-AlSi alloy. NPJH-AlSi samples were solution treated at 500 ℃ for 24 h, followed by an additional joule heating treatment at 800 ℃ for 60 ms. All the tests were measured at 25 ℃ using the 1 M $NaPF_6$ in diglyme =100 vol% electrolyte. Source data for (g and h)are provided as a Source Data file.

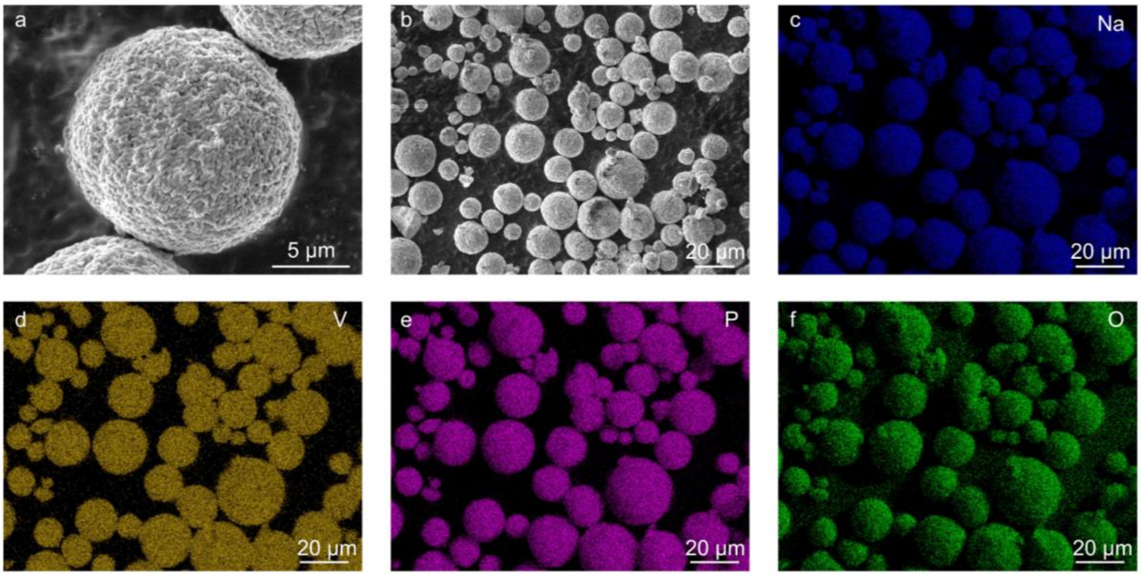

Supplementary Fig. 37. (a, b) SEM images of NVP materials and (c-f) corresponding EDS mappings of Na (blue), V (yellow), P (purple) and O (Green).

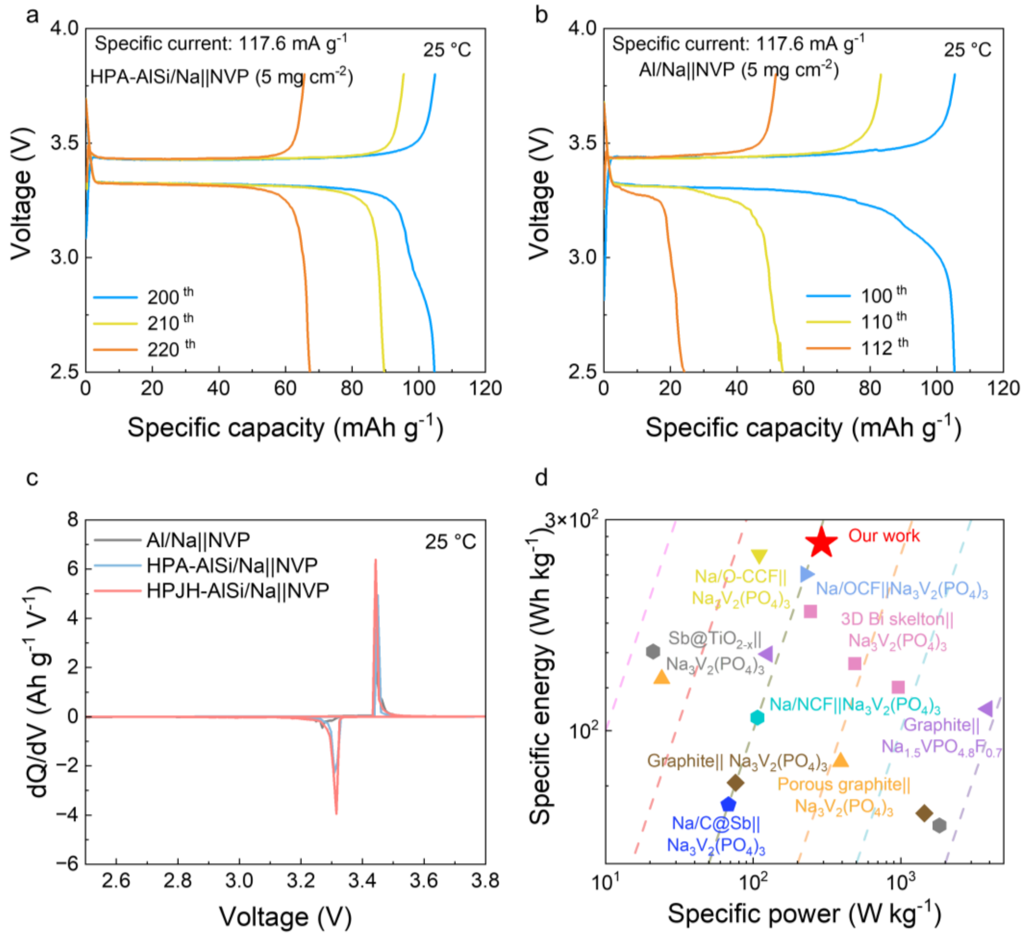

Supplementary Fig. 38. Typical voltage profiles of (a) HPA-AlSi/Na||NVP and (b) Al/Na||NVP cells at the specific current of 117.6 mA g^{-1}. All the tests were measured at 25 ℃. (c) The corresponding dQ/dV plot obtained from the charge/discharge profiles. (d) Comparison of specific energy and specific power between the HPJH-AlSi/Na||NVP cell and previously reported NVP-based full cells. The specific energy and specific power are calculated considering the total mass of positive electrode active materials and deposited Na metal. Source data are provided as a Source Data file.

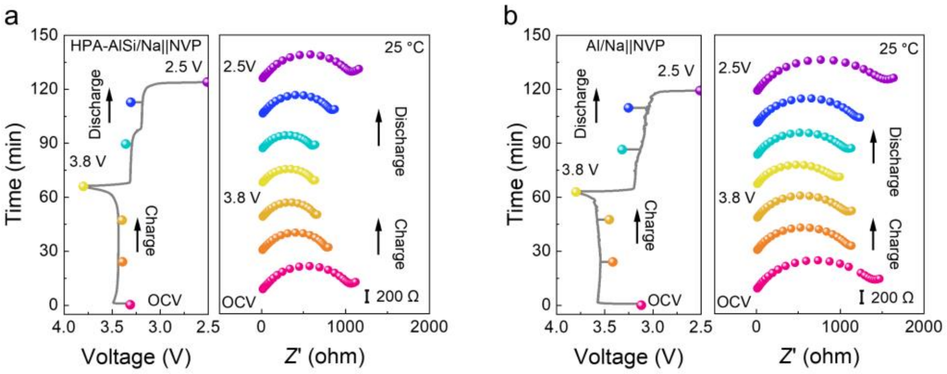

Supplementary Fig. 39. *In situ* EIS results of the (a) HPA-AlSi/Na||NVP and (b) Al/Na||NVP. All the tests were measured at 25 ℃. Source data are provided as a Source Data file.

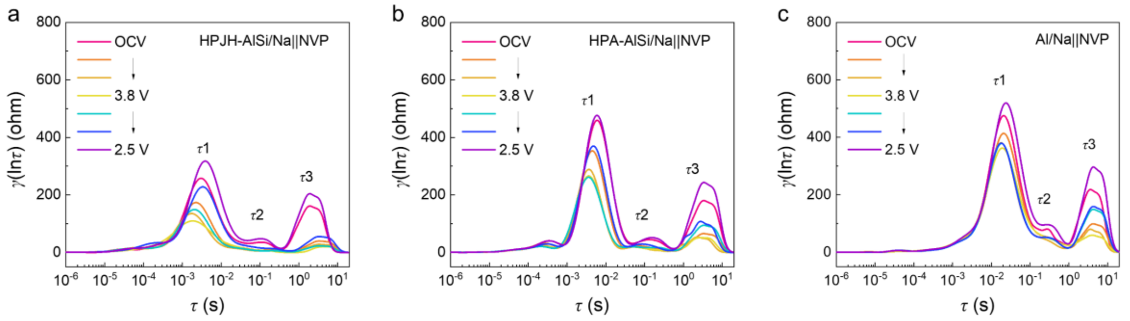

Supplementary Fig. 40. Distribution of Relaxation Times (DRT) calculated from the *in situ* EIS for the discharge and charge process of (a) HPJH-AlSi/Na||NVP, (b) HPA-AlSi/Na||NVP and (c) Al/Na||NVP. All the tests were measured at 25 ℃. Source data are provided as a Source Data file.

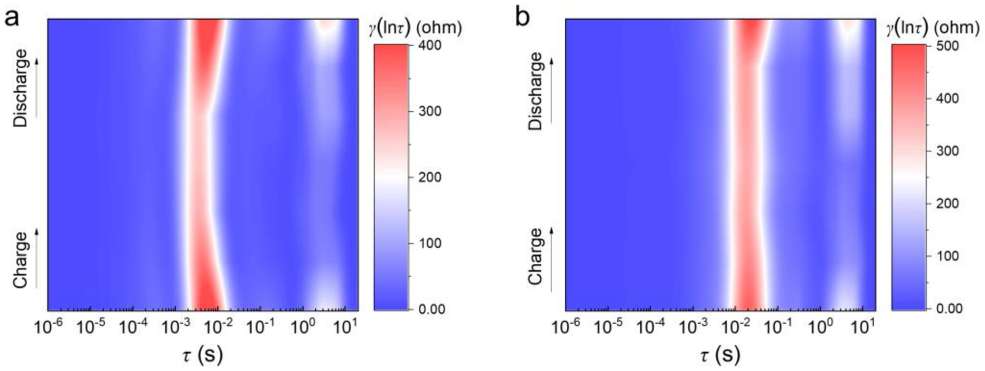

Supplementary Fig. 41. Corresponding contour plots of the calculated DRT results from (a) HPA-AlSi/Na||NVP and (b) Al/Na||NVP. Source data are provided as a Source Data file.

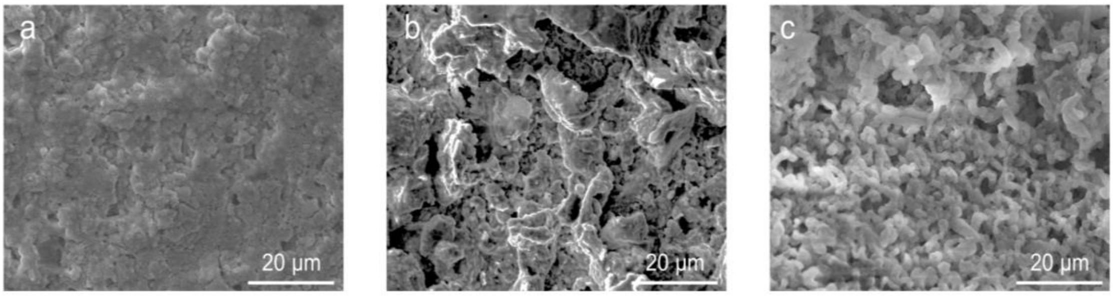

Supplementary Fig. 42. SEM morphologies of negative electrode surface in (a) HPJH-AlSi/Na||NVP, (b) HPA-AlSi/Na||NVP and (c) Al/Na||NVP full cells after Na plating/stripping at a specific current of 117.6 mA g^{-1} for 500 cycles. All the tests were measured under 25 ℃ using the 1 M $NaClO_4$ in DEC:EC=1:1 vol% with 5% FEC electrolyte.

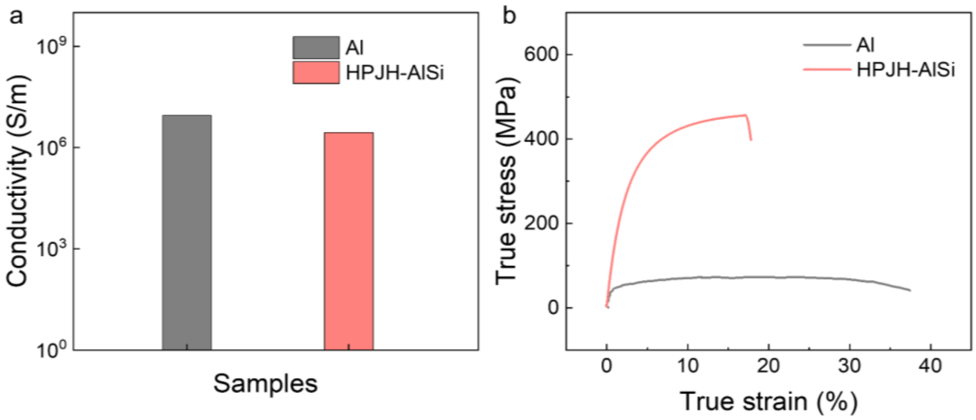

Supplementary Fig. 43. (a) electrical conductivity and (b) mechanical properties of Al and HPJH-AlSi. Source data are provided as a Source Data file.

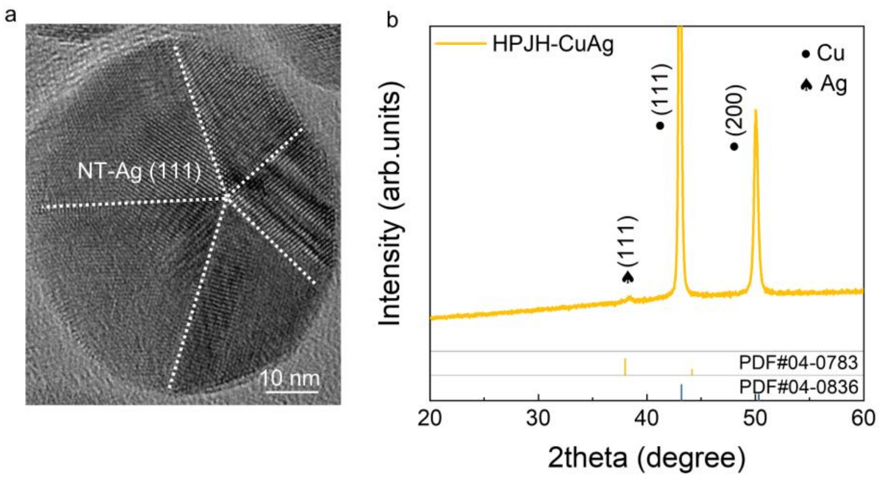

Supplementary Fig. 44. (a) HRTEM images of NT-Ag particle. (b) XRD patterns of the HPJH-CuAg alloy. Source data for (b) are provided as a Source Data file.

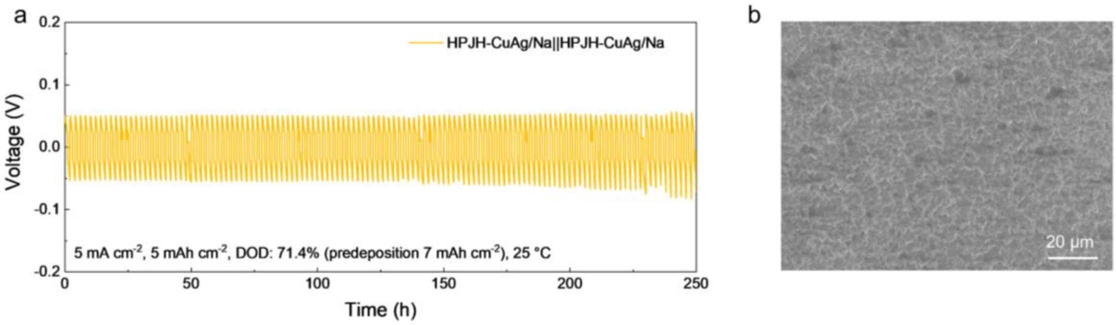

Supplementary Fig. 45. (a) Voltage profiles of symmetric cells assembled with HPJH-CuAg at 5 mA cm^{-2} and 5 mAh cm^{-2}. (b) SEM images of HPJH-CuAg electrodes after Na plating/stripping at 5 mA cm^{-2} and 5 mAh cm^{-2} for 10 cycles. All the tests were measured at 25 ℃ using the 1 M $NaPF_6$ in diglyme =100 vol% electrolyte. Source data for (a) are provided as a Source Data file.

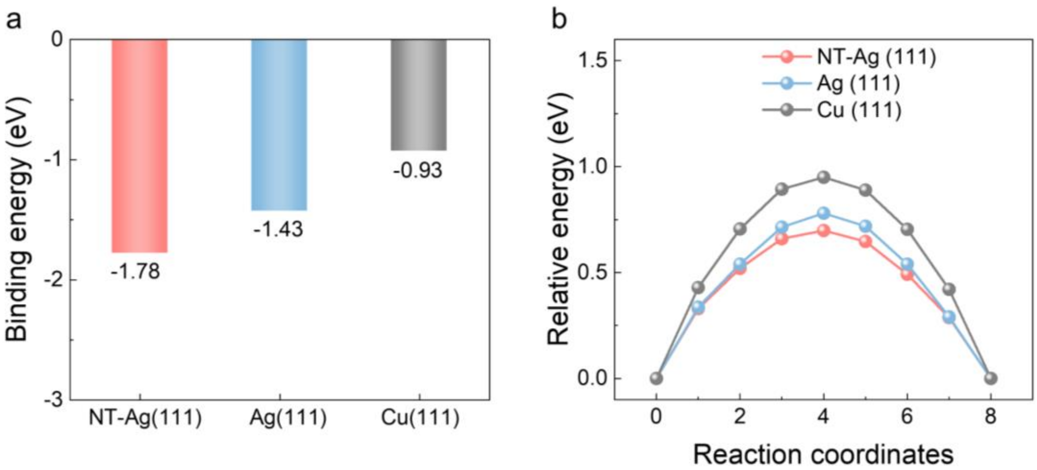

Supplementary Fig. 46. (a) Calculation of the binding energies of different crystal facets with Na atoms. (b) The diffusion energy barrier of Na on the different crystal facets. Source data for (b) are provided as a Source Data file.

Supplementary Note 1

The increase in pressure results in an elevated solubility of Si in Al, and the solidification temperature tends to rise. At a pressure of 5 GPa, the solubility increases to approximately 10 at.% or 10.4 wt.%[2]. Consequently, the low eutectic Al-10 wt. % Si alloy, which is a solid solution alloy at atmospheric pressure, transforms into a supersaturated AlSi solid solution alloy upon solidification at 5 GPa.

Supplementary Note 2

According to the data of the EBSD mapping shown in Supplementary Fig. 8a, it is indicated the samples are mainly composed of equiaxed grains, with uniform size distribution and no preferential orientation. In addition to the IPF analysis, we performed GOS mapping, as shown in Supplementary Fig. 8b. The GOS map provides insight into the internal misorientation of each grain. Grains with GOS values greater than 2 ° are classified as deformed (Supplementary Fig. 8c), while those with values below 2 ° are considered recrystallized[3, 4]. The analysis revealed that 97.1% of the grains in the scanned region had a GOS value of less than 2 °, indicating that the majority of the grains had undergone recrystallization. The KAM mapping is also performed, which highlights local misorientation and provides information about dislocation density and plastic deformation. Supplementary Fig. 8d shows that the KAM distribution, where low KAM values are observed across most grains and grain boundaries, suggesting minimal plastic deformation[5, 6]. The average intra-granular misorientation (θ_{KAM}) is calculated to be approximately 0.2 °, further supporting the conclusion that the grains are largely free of significant deformation.

Supplementary Note 3

The composition of SEI produced on HPJH-AlSi, HPA-AlSi and Al collectors after deposition is mainly dominated by NaF, Na_2CO_3 and Na_2O. In addition, the compositions of the SEI films on the HPJH-AlSi, HPA-AlSi and Al collectors remained basically the same, indicating that the differences in morphology observed in SEM are not related to the SEI film compositions, and it is the collector compositions that determine the variations in the deposition morphology.

Supplementary Note 4

To enhance the visualization of porosity variations and their evolution with increasing current or capacity, we present a summary of the thickness data for the deposited Na layer under various current densities and capacities in Supplementary Fig. 10, 12. The data consistently demonstrate that, irrespective of the applied current or capacity, the Na plating on the HPJH-AlSi consistently exhibits significantly higher density.

The black dotted line in the figures denote the thickness of fully dense Na deposits. Specifically, the thickness of deposited Na for 1 mAh cm^{-2} capacity is 8.9 μm, which is calculated as follows:

$$Thickness = \frac{CM}{nF} * \frac{1}{\rho} \qquad (1)$$

where C represents the areal capacity (mAh cm^{-2}), M stands for the molar mass (g mol^{-1}), n signifies the number of electrons transferred, F denotes the Faraday constant (C mol^{-1}), and ρ indicates the density (g cm^{-3}). Consequently, for a deposition capacity of 1 mAh cm^{-2}, the associated thickness calculated approximates 8.9 μm.

Supplementary Note 5

The deposition morphology on a substrate is determined by the relative magnitudes of the electrochemical reaction rate and the surface diffusion rate[7, 8]. For Na deposition on various substrates, these parameters are related to the experimental current density and the self-diffusion coefficient of Na. The electrochemical reaction rate, k_e, can be derived from the experimental current density, J, as follows:

$$k_e = \frac{Ja^2}{F} N_A \qquad (2)$$

Where a is the size of one lattice cell, F is Faraday's constant, and N_A is Avogadro's constant. The values of these parameters are listed in Supplementary Table 1. This paper introduces three primary current densities: 1, 3 and 5 mA cm^{-2}, respectively. Correspondingly, the k_e values are 22.47, 67.41 and 112.34 s^{-1}, respectively.

The surface diffusion rate, k_d, is calculated using Equation S3 and is dependent on the activation energy barrier for self-diffusion, E_{diff}, temperature,T, and jump frequency for Na diffusion, v. The literature reports average values of jump frequency, which range between ca. 10^{12}-10^{13} s^{-1}. Therefore, a value of $v = 5\times10^{12}$ s^{-1} has been utilized where k_B represents the Boltzmann constant.

$$k_d = v\, exp\left(\frac{-E_{diff}}{k_B T}\right) \qquad (3)$$

The self-diffusion energy barrier, E_{diff}, determines the magnitude of the diffusion rate and therefore must be accurately measured to capture the underlying dynamics of the Na system. In this paper, we used DFT calculations to determine the surface Na diffusion barriers of NT-Si, Si, and Al, which are 0.86, 0.62, and 0.48 eV, respectively. To simplify the calculations, we consider the corresponding diffusion rates for the three types of collectors (Al, HPA-AlSi and HPJH-AlSi) to be 1.38×10^{5}, 1.92×10^{2}, and 1.78×10^{-2} s^{-1}.

Supplementary Note 6

Initially, the increase in current reflects the expansion of the electroactive area, attributable to the growth in size of existing nuclei and the generation of new nuclei on the surface[9]. Subsequently, the current diminishes and converges to a stable state as a result of the overlapping of adjacent nuclei and their diffusion zones, signifying a growth process[10].

Supplementary Note 7

The cumulative capacity of the symmetrical cell was calculated using the following formula[11]: Cumulative capacity = Capacity per cycle × Cycle number, in units of Ah cm^{-2}.

Supplementary Note 8

We measured the time of coating shedding on the collector surface at different ultrasonic powers, and we can find that the coating on Si-coated-Al is easy to shed, which causes the coating to separate from the collector during the Na plating/stripping process, resulting in non-uniform deposition of the collector.

Supplementary Note 9

The dQ/dV results reveal oxidation and reduction peaks that align well with the voltage platforms of Na plating and extraction in $Na_3V_2(PO_4)_3$ (NVP) cells. No characteristic peaks related to the alloying reactions of Al or Si with Na are detected. Moreover, the HPJH-AlSi/Na||NVP exhibits the largest peak intensity and the lowest polarization compared to Al/Na||NVP and HPA-AlSi/Na||NVP, indicating that HPJH-AlSi/Na retains the highest active Na content [12, 13]. Additionally, only in the Al/Na||NVP cell is a reduction peak corresponding to the dissolution of bulk Na^+ observed, whereas no such peak is detected in the HPJH-AlSi/Na||NVP cell. This indicates the presence of a significant amount of inactive Na in the Al/Na||NVP cell[12]. In contrast, due to the fast Na dynamics in the HPJH-AlSi, uniform Na deposition and stripping are achieved, significantly reducing the formation of inactive Na.

Supplementary Note 10

The evaluation of specific energy (E) and specific power (P) relies on the total mass of the active electrodes, as expressed by the following equations

$$E = \frac{VCm_{NVP}}{m_{NVP}+m_{Na}} \qquad (4)$$

where V represents the average discharge voltage and C denotes the specific capacity, m_{NVP} and m_{Na} correspond to the masses of the NVP and the host Na, respectively.

Specific power (P) is determined using the following equation[14]:

$$P = \frac{EI}{C} \qquad (5)$$

In this equation, where I represents the specific current density.

Supplementary Note 11

The DRT method has been utilized for the precise quantification of individual resistance components, avoiding any artificial fitting errors. Fundamentally, it constitutes a mathematical transformation wherein the frequency domain Nyquist plots (Supplementary Fig. 39) are converted into the time domain DRT profile using following equation[15, 16, 17]:

$$Z_{DRT} = R_{\infty} + \int_{-\infty}^{\infty} \frac{\gamma(ln\tau)}{1+i2\pi} dln\tau \qquad (6)$$

In which, every physical process is depicted as a local maximum within a continuous distribution function[17], and the corresponding impedance value is quantified by the area under the peak[18].

Supplementary Note 12

Regarding electrical conductivity tests: The resistivity of the material is derived using the van der Pauw method (vdp). This four-probe method is applied to small, flat samples with four terminals and uniform thickness. A current is applied through two terminals of the sample, and the voltage drop is measured across the opposite two terminals. The specific formula is as follows:

$$Q_A = \left(\frac{R_{12,43}^{+} - R_{12,43}^{-}}{R_{23,14}^{+} - R_{23,14}^{-}}\right) = \left(\frac{V_{12,43}^{+} - V_{12,43}^{-}}{I_{12}^{+} - I_{12}^{-}}\right)\left(\frac{I_{23}^{+} - I_{23}^{-}}{V_{23,14}^{+} - V_{23,14}^{-}}\right)$$

$$Q_B = \left(\frac{R_{34,21}^{+} - R_{34,21}^{-}}{R_{41,32}^{+} - R_{41,32}^{-}}\right) = \left(\frac{V_{34,21}^{+} - V_{34,21}^{-}}{I_{34}^{+} - I_{34}^{-}}\right)\left(\frac{I_{41}^{+} - I_{41}^{-}}{V_{41,32}^{+} - V_{41,32}^{-}}\right)$$

$$\frac{Q-1}{Q+1} = \frac{f}{ln2}\cos h^{-1}\left\{\frac{1}{2}exp\left[\frac{ln2}{f}\right]\right\}$$

$$R_A = \frac{\pi \cdot f_A}{ln2}\left\{\frac{V_{12,43}^{+} - V_{12,43}^{-} + V_{23,14}^{+} - V_{23,14}^{-}}{I_{12}^{+} - I_{12}^{-} + I_{23}^{+} - I_{23}^{-}}\right\}$$

$$R_B = \frac{\pi \cdot f_B}{ln2}\left\{\frac{V_{34,21}^{+} - V_{34,21}^{-} + V_{41,32}^{+} - V_{41,32}^{-}}{I_{34}^{+} - I_{34}^{-} + I_{41}^{+} - I_{41}^{-}}\right\}$$

$$\rho_A = R_A \cdot t$$

$$\rho_B = R_B \cdot t$$

$$\rho_{av} = \frac{\rho_A + \rho_B}{2}$$

Here, R is the sample resistance, A is the test current, V is the voltage, and ρ is the resistivity. t is the thickness of the sample and f is the geometric factor of the symmetry of the sample which is related to the two resistance ratios Q.

Finally, the reciprocal of the calculated resistivity is taken to obtain the electrical conductivity.

Regarding mechanical performance tests: Dog-bone tensile test samples with a gauge of 8 mm were used in tensile tests. The tensile tests were performed at room temperature at aninitial strain rate of 1.0×10^{-3} s^{-1}. The value was averaged over at least three measurements.

Supplementary Table 1. Model parameters.

Parameters		Values	Units
a	Lattice cell dimension	0.6	nm
J	Average current density	1,3,5	mA cm^{-2}
F	Faraday's constant	96487	C/mol
N_A	Avogadro's constant	6.022×10^{23}	L/mol
T	Operating temperature	300	K
v	Vibration frequency	5×10^{12}	s^{-1}
k_B	Boltzmann's constant	8.617×10^{-5}	eV K^{-1}

Supplementary Table 2. Summary of depth of discharge in Na symmetric cells in recent research on Na metal batteries.

	Cumulative capacity (Ah cm^{-2})	Cycle time (h)	Current density (mA cm^{-2})	Areal capacity per cycle (mAh cm^{-2})	DOD Na (%)	Reference
Porous Al	0.25	1000	0.5	0.25	12.5	[19]
Na-AgNP	0.4	800	1	1	25	[20]
Co-NC-Cu	0.6	1200	1	1	33.33	[21]
Bi-CNs-Cu	0.695	1390	1	3	50	[22]
	1.4	2800	1	2	33.3	
	2	4000	1	1	16.67	
3D Zn@Al	0.75	1500	2	1	50	[23]
C@Ag-Cu	0.8	1600	1	4	66.67	[24]
	1.8	3600	1	3	50	
C@Sb-Cu	1.2	2400	1	1	25	[25]
Cu_3P@Cu	2	2000	2	2	-	[26]
At-Sn@HCN-Cu	4.5	1800	5	5	50	[27]
	2.5	5000	1	3.4	85	
	10	5000	4	8	80	
PAN/Sn	2.5	2500	2	2	40	[28]
FCTF-Cu	2.6	1300	4	9.5	95	[29]
	2.6	2600	2	1	50	
Na@PZC	6.25	2500	5	5	-	[30]
SnNCNF-Cu	7.5	5000	3	3	26	[31]
	9.75	3920	5	5	43	
O_f-CNT	8.75	3500	5	1	-	[32]

Our work	8.4	5600	3	3	60	-
Our work	13.25	5300	5	5	71.4	
Our work	3.2	800	8	8	80	-

Supplementary Table 3. Summary of electrochemical properties of five types of collectors prepared in our work.

	Average CE value (%)	CE cycle number (n)	CE polarization (mV)	Symmetrical battery cycle time (h)	Lowest battery overpotential (mV)
HPJH-AlSi	99.71	4000	43.7	5300	23
HPA-AlSi	98.38	1500	44.0	1280	30
As-cast-AlSi	96.22	275	48.3	30	46
HP-AlSi	95.69	100	80.6	25	51
Al	94.46	40	141.6	24	53

Supplementary Table 4. Summary of NVP-based full cells in recent Na negative electrode research studies.

Negative electrode materials	N/P ratio	Mass loading (mg/cm^2)	Specific current ($mA\ g^{-1}$)	Cycle numbers (n)	Capacity retention (%)	Reference
OCF	4.25	2	117.6	200	97.5	33
CT-Sn(II)@Ti_3C_2	12.7	2	117.6	200	91.8	34
Sn/C@Cu	25.5	2	117.6	100	96.5	35
C@Sb@Cu	25.5	2	117.6	500	95	25
NCF	26-28	2.4-2.6	117.6	300	83	36
MgF_2@RGO	18.9	2	117.6	200	91.3	37
r-GO	5	10	117.6	25	60.8	38
Al-Cu@C	4.3-4.9	4	117.6	400	94	39
Al-Cu@C	10.6-12.2	3.5-4	117.6	1300	93	39
Our work	3.7	5	117.6	500	95.4	-

Supplementary Table 5. Summary of NVP-based full cells in recent Na negative electrode research studies.

Full cell	Specific power (W kg^{-1})	Specific energy (Wh kg^{-1})	Reference
3D Bi skeleton|| $Na_3V_2(PO_4)_3$	244.7	186	[40]
	488.9	141.8	
	964.2	125.3	
Sb@TiO_{2-x}||$Na_3V_2(PO_4)_3$	21	151	[41]
	1830	61	
Porous graphite||$Na_3V_2(PO_4)_3$	24	131	[42]
	392	85	
Graphite||$Na_3V_2(PO_4)_3$	76	76.2	[43]
	1444	65	
Graphite||$Na_{1.5}VPO_{4.8}F_{0.7}$	125	149	[44]
	3863	112	
Na/C@Sb||$Na_3V_2(PO_4)_3$	68	68	[25]
Na/NCF||$Na_3V_2(PO_4)_3$	107	107	[36]
Na/O-CCF||$Na_3V_2(PO_4)_3$	110	250	[45]
Na/OCF||$Na_3V_2(PO_4)_3$	226	226	[33]
Our work	290.55	265.85	

Supplementary Table 6. Summary of electrical conductivity and mechanical properties of Al and HPJH-AlSi.

Samples	Electrical conductivity (S/m)	Yield strength (MPa)	Tensile strength (MPa)	Elongation rate (%)
Pure Al	8.94×10^{6}	45	72	37
HPJH-AlSi	2.77×10^{6}	344	456	17

References

1. Sun, J., et al. Lithium deposition mechanism on Si and Cu substrates in the carbonate electrolyte. *Energy Environ. Sci.* **15**, 5284-5299 (2022).
2. Yu, X., et al. Non-equilibrium microstructure of hyper-eutectic Al-Si alloy solidified under superhigh pressure. *J. Mater. Sci.* **34**, 4149-4152 (1999).
3. Yang, Y., et al. On the micromechanism of superior strength and ductility synergy in a heterostructured Mg-2.77 Y alloy. *J. Magnes. Alloy* **12**, 2793–2811 (2024).
4. Wu, S., et al. Hot deformation behavior and microstructure evolution of a novel Al-Zn-Mg-Li-Cu alloy. *Materials* **15**, 6769 (2022).
5. Zhang, K., et al. A comparative study of plastic deformation mechanisms in room-temperature and cryogenically deformed magnesium alloy AZ31. *Mater. Sci. Eng. A* **807**, 140821 (2021).
6. Shen, R. R. & Efsing, P. Overcoming the drawbacks of plastic strain estimation based on KAM. *Ultramicroscopy* **184**, 156-163 (2018).
7. Hao, F., Verma, A. & Mukherjee, P. P. Mesoscale complexations in lithium electrodeposition. *ACS Appl. Mater. Inter.* **10**, 26320-26327 (2018).
8. Davidson, R., et al. Formation of magnesium dendrites during electrodeposition. *ACS Energy Lett.* **4**, 375-376 (2018).
9. Trejo, G., et al. Nucleation and growth of zinc from chloride concentrated solutions. *J. Electrochem. Soc.* **145**, 4090 (1998).
10. Scharifker, B. & Hills, G. Theoretical and experimental studies of multiple nucleation. *Electrochim. Acta* **28**, 879-889 (1983).
11. Liu, W., et al. Lithium-activated SnS–graphene alternating nanolayers enable dendrite-free cycling of thin sodium metal anodes in carbonate electrolyte. *Energy Environ. Sci.* **14**, 382-395 (2021).
12. Luo, Z., et al. Robust artificial interlayer for columnar sodium metal anode. *Nano Energy* **97**, 107203 (2022).
13. Jiao, W., et al. Highly stable anode-free sodium batteries enabled by mechanically deformable nucleation interface. *Energy Storage Mater.* **73**, 103784 (2024).
14. Li, S., et al. Space-confined guest synthesis to fabricate Sn-monodispersed N-doped mesoporous host toward anode-free Na batteries. *Adv. Mater.* **35**, 2301967 (2023).
15. Illig, J., Ender, M., Weber, A. & Ivers-Tiffée, E. Modeling graphite anodes with serial and transmission line models. *J. Power Sources* **282**, 335-347 (2015).

16. Illig, J., et al. Understanding the impedance spectrum of 18650 $LiFePO_4$-cells. *J. Power Sources* **239**, 670-679 (2013).
17. Schmidt, J. P., et al. The distribution of relaxation times as basis for generalized time-domain models for Li-ion batteries. *J. Power Sources* **221**, 70-77 (2013).
18. Lu, Y., et al. The carrier transition from Li atoms to Li vacancies in solid-state lithium alloy anodes. *Sci. Adv.* **7**, eabi5520 (2021).
19. Liu, S., et al. Porous Al current collector for dendrite-free Na metal anodes. *Nano Lett.* **17**, 5862-5868 (2017).
20. Wang, Z., et al. Lightweight, thin, and flexible silver nanopaper electrodes for high-capacity dendrite-free sodium metal anodes. *Adv. Funct. Mater.* **28**, 1804038 (2018).
21. Xie, Y., et al. Encapsulating sodium deposition into carbon rhombic dodecahedron guided by sodiophilic sites for dendrite-free Na metal batteries. *Energy Stor. Mater.* **30**, 1-8 (2020).
22. Zhang, L., et al. Bi nanoparticles embedded in 2D carbon nanosheets as an interfacial layer for advanced sodium metal anodes. *Small* **17**, 2007578 (2021).
23. Cai, Z., et al. A multifunctional super-sodiophilic coating on aluminum current collector for high-performance anode-free Na-metal batteries. *Nano Energy* **116**, 108814 (2023).
24. Zhu, N., et al. Stable sodium metal anodes with a high utilization enabled by an interfacial layer composed of yolk-shell nanoparticles. *J. Mater. Chem. A* **9**, 13200-13208 (2021).
25. Wang, G., et al. Core-shell C@Sb nanoparticles as a nucleation layer for high-performance sodium metal anodes. *Nano Lett.* **20**, 4464-4471 (2020).
26. Zhang, W., et al. Anode-free sodium metal pouch cell using Cu_3P nanowires in situ grown on current collector. *Adv. Mater.* **36**, 2310347 (2024).
27. Xu, F., et al. Atomic Sn-enabled high-utilization, large-capacity, and long-life Na anode. *Sci. Adv.* **8**, eabm7489 (2022).
28. Xu, Y., et al. Sodium deposition with a controlled location and orientation for dendrite-free sodium metal batteries. *Adv. Energy Mater.* **10**, 2002308 (2020).
29. Zhuang, R., et al. Fluorinated porous frameworks enable robust anode-less sodium metal batteries. *Sci. Adv.* **9**, eadh8060 (2023).
30. Bai, W., et al. Sodiophilic three-dimensional carbon skeleton derived from polyacrylonitrile@ zeolitic imidazolate framework fiber for dendrite-free sodium

metal anode. *J. Power Sources* **551**, 232165 (2022).

31. Li, S., et al. Space-confined guest synthesis to fabricate Sn-monodispersed N-doped mesoporous host toward anode-free Na batteries. *Adv. Mater.* **35**, 2301967 (2023).
32. Ye, L., et al. A sodiophilic interphase-mediated, dendrite-free anode with ultrahigh specific capacity for sodium-metal batteries. *Angew. Chem. Int. Ed.* **131**, 17210-17216 (2019).
33. Cui, X. Y., et al. A carbon foam with sodiophilic surface for highly reversible, ultra-long cycle sodium metal anode. *Adv. Sci.* **8**, 2003178 (2021).
34. Luo, J., et al. Pillared MXene with ultralarge interlayer spacing as a stable matrix for high performance sodium metal anodes. *Adv. Funct. Mater.* **29**, 1805946 (2019).
35. Wang, G., et al. 2D Sn/C freestanding frameworks as a robust nucleation layer for highly stable sodium metal anodes with a high utilization. *Nano Energy* **79**, 105457 (2021).
36. Liu, B., et al. 3D uniform nitrogen-doped carbon skeleton for ultra-stable sodium metal anode. *Nano Res.* **13**, 2136-2142 (2020).
37. Zhao, L., et al. In situ plating of Mg sodiophilic seeds and evolving sodium fluoride protective layers for superior sodium metal anodes. *Adv. Energy Mater.* **12**, 2200990 (2022).
38. Wang, A., et al. Processable and moldable sodium-metal anodes. *Angew. Chem. Int. Ed.* **129**, 12083-12088 (2017).
39. Li, H., et al. Sodiophilic current collectors based on MOF-derived nanocomposites for anode-less Na-metal batteries. *Adv. Energy Mater.* **12**, 2202293 (2022).
40. Zhang, J., et al. The origin of anode–electrolyte interfacial passivation in rechargeable Mg-metal batteries. *Energy & Environmental Science* **16**, 1111-1124 (2023).
41. Wang, N., Bai, Z., Qian, Y. & Yang, J. Double-walled Sb@TiO_{2-x} nanotubes as a superior high-rate and ultralong-lifespan anode material for Na-ion and Li-ion batteries. *Adv. Mater.* **28**, 4126-4133 (2016).
42. Han, P., et al. Flexible graphite film with laser drilling pores as novel integrated anode free of metal current collector for sodium ion battery. *Electrochem. Commun.* **61**, 84-88 (2015).
43. Zhu, Z., et al. Highly stable and ultrafast electrode reaction of graphite for sodium ion batteries. *J. Power Sources* **293**, 626-634 (2015).

44. Xu, Z. L., et al. Tailoring sodium intercalation in graphite for high energy and power sodium ion batteries. *Nat. Commun.* **10**, 2598 (2019).

45. Li, T., et al. Superior sodium metal anodes enabled by sodiophilic carbonized coconut framework with 3D tubular structure. *Adv. Energy Mater.* **11**, 2003699 (2021).
