# Supplementary material for: A nanotwinned-alloy strategy enables fast sodium deposition dynamics
Source: Nat Commun. 2025 Feb 20;16:1795. doi: 10.1038/s41467-025-56957-w (PMC11842702; doi:10.1038/s41467-025-56957-w)
Supplement: Supplementary file 3 — Description of Additional Supplementary Files [file 41467_2025_56957_MOESM3_ESM.pdf]

Description of Additional Supplementary Files

Supplementary Movie 1: In situ TEM images of Na metal deposition on the HPJH-AlSi collector.

Supplementary Movie 2: In situ TEM images of Na metal deposition on the HPA-AlSi collector.

Supplementary Movie 3: In situ TEM images of Na metal deposition on the Al collector.
